# The Complex Genetic Architecture of Early Root and Shoot Traits in Flax Revealed by Genome-Wide Association Analyses

**DOI:** 10.3389/fpls.2019.01483

**Published:** 2019-11-19

**Authors:** Demissew Sertse, Frank M. You, Sridhar Ravichandran, Sylvie Cloutier

**Affiliations:** ^1^Department of Biology, University of Ottawa, Ottawa, ON, Canada; ^2^Ottawa Research and Development Center, Agriculture and Agri-Food Canada, Ottawa, ON, Canada

**Keywords:** flax, root, shoot, genome-wide association study, quantitative trait nucleotides, candidate genes

## Abstract

Roots are fundamental organs for water and nutrient uptake as well as for signal transduction in response to biotic and abiotic stresses. Flax has a shallow tap root system that relies mostly on top soil nutrient and moisture resources. The crop can easily be outcompeted by weeds or other crops in intercropping systems, especially in moisture deficit conditions. However, there is a wide range of variation among genotypes in terms of performance under scarce resources such as moisture limitation. Here we phenotyped 15 root, two shoot traits and shoot to root dry weight ratio on 115 flax accessions grown in a hydroponic pouch system and performed a genome-wide association study (GWAS) based on seven different models to identify quantitative trait loci underlying these traits. Significant variation among genotypes was observed for the two shoot and 12 of the 14 root traits. Shoot dry weight was correlated with root network volume, length, surface area, and root dry weight (*r* > 0.5, *P* < 0.001) but not significantly correlated with root depth (*r* = 0.033, *P* > 0.05). The seven GWAS models detected a total of 228 quantitative trait nucleotides (QTNs) for 16 traits. Most loci, defined by an interval of 100 kb up and downstream of the QTNs, harbored genes known to play role(s) in root and shoot development, suggesting them as candidates. Examples of candidate genes linked to root network QTNs included genes encoding GRAS transcription factors, mitogen-activated protein kinases, and auxin related lateral organ boundary proteins while QTN loci for shoot dry weight harbored genes involved in photomorphogenesis and plant immunity. These results provide insights into the genetic bases of early shoot and root development traits in flax that could be capitalized upon to improve its root architecture, particularly in view of better withstanding water limiting conditions during the cropping season.

## Introduction

Roots are vital organs in terrestrial higher plants for acquisition of essential nutrients and water. Because roots function in a bio-physico-chemically dynamic rhizosphere, they play an important role in controlling and regulating the impacts of various edaphic factors through internal physiological adjustments (Jackson et al., 1990; Hodge 2004) and signal transduction (Batool et al., 2018). Adaptation of plants to a scarcity of resources and associated edaphic factors is therefore governed by their root system. The architectural features of root systems are crucial in efficiently tapping the available resources such as water and nutrients in the rhizosphere (Yue et al., 2006) and inadequate root system development may lead to significant yield losses in water-limiting conditions (Henry et al., 2011). Understanding root traits and resource use efficiencies of the root system is key to crop yield improvement (Kell, 2011). However, the inaccessibility of the rhizosphere has made root trait studies challenging; hence these traits have been scantly considered in varietal improvement.

Flax, one of the founder crops of agriculture (Weiss and Zohary, 2011), has been grown as both fiber and oilseed crops for nearly the entire span of its cultivation history (Herbig and Maier, 2011). The crop is adapted to diverse ecologies, from the warm Indian subcontinent to the cool temperate areas in Eurasia (Casa et al., 1999; Sertse et al., 2019). A wide range of uses are derived from its stem fibers and its oil-rich seeds (Singh et al., 2011). Flax production, however, is constrained by low yield (Wittkop et al., 2009). The meager flax yield improvements of ∼0.5 ton/Ha since the 1960s, obtained through breeding and agronomic practices, have not been sufficient to impact its production in a major way and production continues to decline as growers are shifting to better yielding crops such as soybean, canola (http://www.fao.org/faostat/en/#data). High and stable yielding cultivars are urgently needed to rekindle growers' interest and meet market potential.

Flax, like other oilseed crops, is a tap-rooted plant. Compared to canola, sunflower, and safflower, flax has a shallower root system and, as such, it mainly relies on moisture and nutrient resources available in the soil's top layers (Hocking et al., 1997; Kar et al., 2007) mainly within 70 cm depth ((Flax Council of Canada, 2015; Hall et al., 2016). Flax roots can grow to depth of 90–120 cm with a lateral spread of ∼ 30 cm in light soil (Gill 1987). However, the proportion of its root deeper than 60 cm is only 4–7% (Hall et al., 2016) and the roots rarely grow beyond 80 cm (Flax Council of Canada, 2015). Unlike other crops such as canola, mustard and wheat that have aggressive root growth before early flowering the fastest root growth in flax is between early flowering to late flowering where it declines late bolling stage onwards (Liu et al., 2011). Under limited water, flax can be easily outcompeted by many weeds, exacerbating the competition for resources (Bell and Nalewaja, 1968; Gruenhagen and Nalewaja, 1969; Alessi and Power, 1970; Klimek-Kopyra et al., 2015). Flax is also less competitive than fibrous rooted cereals such as wheat (Morillon and Lassalles, 2002) that are with more extensive roots than flax in top soil layer (Gill, 1987). Despite the fact that flax performed well being intercropped with chickpea in irrigated field when flax grown in the furrow and chickpea on the ridge, poor flax performance was noticed when both the crop grown on flat field suggesting the low competence of flax (Ahlawat and Gangaiah, 2010). However, a wide range of performance among flax genotypes under different moisture regimes (Foster et al., 1998; Diederichsen et al., 2006) can be attributed to variations of their root system (Cattivelli et al., 2008).

Prompted by the current large sets of genetic data from genome-wide association studies (GWAS) and advancements in imaging and data processing, high throughput digital root phenotyping techniques have become attractive. Hund et al. (2009) used a pouch system to phenotype root traits in maize and, this system has subsequently been applied in other crops such as wheat (Atkinson et al., 2015), Brassica (Thomas et al., 2016), and barley (Canto et al., 2018) for examples.

Here we applied this technique to study the early root and shoot development of a flax mini-core collection (n = 115) that comprises representative genotypes from all major flax growing regions of the world. A GWAS was performed using a set of single nucleotide polymorphism (SNP) markers obtained from shotgun short-read re-sequencing data of the germplasm collection. The objectives of this research were 1) to assess the extent of the variation in root traits among genotypes, 2) to identify quantitative trait nucleotides (QTNs) associated with the genetic architecture of selected root and shoot traits, and, 3) to identify candidate genes for the traits harbored at the QTN loci.

## Materials and Methods

### Plant Materials

A flax mini-core collection (n = 115) that comprised >95% of the genetic diversity (Soto-Cerda et al., 2013) of the flax core collection (n = 407) (Diederichsen et al., 2013) was used. The 75 linseed, 33 fiber flax, and 7 accessions of unknown type of the mini-core collection were collected from 20 countries that represent all major flax growing regions of the world.

### Phenotyping

Early development root and shoot phenotyping was performed in a hydroponic pouch system modified from Hund et al. (2009). The system comprised two large plastic bins each containing 108 L of nutrient solution that was transferred every 3 h from one bin to the other using timer-operated peristaltic pumps. Aluminum frames mounted on top of the two opaque bins were used to hang the pouches. The bins, but not the plants, were covered with black polyethylene sheets to prevent algal growth. This system was installed in a growth chamber (Conviron PGC20, Serial No. 150342, Controlled Environment Ltd, Canada) maintained at 21/18°C with a 16 h day/8 h night photoperiod.

**Figure 1:**
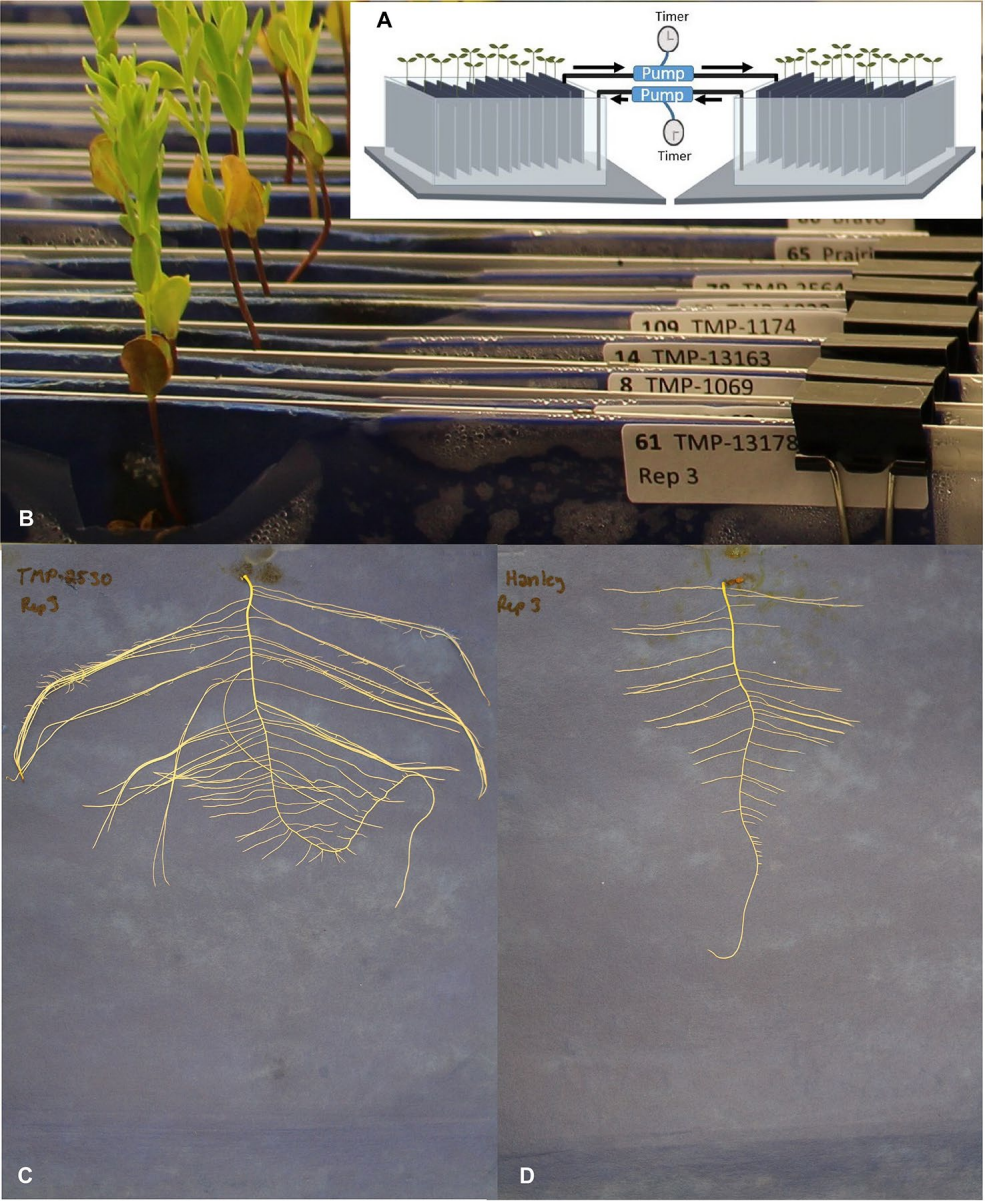
Early root phenotyping experiment set-up and representative root images. **(A)** Diagrammatic sketch of the phenotyping system, arrows show the water flow direction, **(B)** close-up of the experimental set-up showing a partial view of the upper section of the blotting papers, labels, and plantlets, **(C)** extensive root system of TMP-2530 (U_MAR_C_CN98193), and **(D)** root system of flax cultivar Hanley.

Sterile 24x30 cm blue germination blotting papers (SGB1924B, Anchor Paper Company, St Paul, MN, USA) were inserted in Ziploc bags. The assemblies were attached to rust-proofed rods using fold back clips that were suspended in the bins with the open side of the Ziploc bags down to allow blotting up of the nutrient solution. Assemblies were labeled and randomized for each of the three consecutive biological replicates performed (**Figure 1B**).

The Hoagland nutrient solution (HOP1, Hoagland's No. 2 basal salt mixture, Caisson Labs, Smithfield, UT, USA) was made at 25% strength with deionized water and adjusted to pH∼6.3. The pouches were randomly distributed in the two bins. After complete moistening of the blotting paper, three seeds were inserted directly in the paper using tweezers, ∼2 cm below the top edge and slightly spaced out near the center.

The siphons that transferred the nutrient solution were set ∼3 cm from the bottom of the bins, i.e., touching the bottom of the blotting papers, in order to keep them moist at all time. Five days after seeding, germinated seeds were thinned out to one seedling per pouch. The retained seedlings were allowed to grow for 18 days.

On the 19^th^ day, plants were carefully removed from their assemblies for imaging and measurements. Shoots, cut at the shoot and root junction, were measured with a ruler to estimate shoot length prior to being transferred to labeled envelopes and dried for 3 days at 60°C to measure dry weight. The root system of each plant was imaged using a Canon camera (EOS Rebel T5i) mounted on a custom stand to ensure a consistent 40 cm distance between lens and roots. After imaging, the roots were gently pealed from the paper and processed as the shoots to measure their dry weight. Three biological replicates were thus performed consecutively.

Root images were processed using the General Image Analysis of Roots (GIA Roots) software (Galkovskyi et al., 2012). Each image was scaled using the 30 cm edge of the paper as reference. Scaled images were then analyzed for 14 root traits (**Table 1**, see details of the traits at http://www.rootnet.biology.gatech.edu/giaroots/download/recent/gia_roots_manual.pdf). 

**Table 1:** Phenotypic traits and their basic statistics of three replicates of the 111 accessions of the flax mini-core collection.

Trait	Abbreviation	Description	Unit^1^	Range	Median	Mean ± SD^2^
Average root diameter	ARD	Average individual root diameter	cm	0.01–0.04	0.03	0.035 ± 0.0001**
Maximum number of roots	MaxR	After sorting the number of roots crossing a horizontal line from smallest to largest, the maximum number is considered to be the 84th-percentile value (one standard deviation)	count	2–19	8.33	8.94 ± 3.37**
Median number of roots	MedR	The result of a vertical line sweep in which the number of roots that crossed a horizontal line was estimated, and then the median of all values for the extent of the network was calculated.	count	1–10	3.67	3.99 ± 1.65**
Network area	NWA	Total area covered by all roots	cm^2^	1.92–14.67	7.31	7.27 ± 2.15**
Network depth	NWDep	The maximum depth reached by the root	cm	10.95–24.91	20.29	20.04 ± 2.51
Network distribution	NWDist	The fraction of root network in the lower two third of the network (analogy of root depth density)	na	0.26–1.98	0.79	0.865 ± 0.331
Network length	NWL	Total length of the entire network (∼half perimeter)	cm	156.2–513.7	255.9	245.3 ± 77.5**
Network perimeter	NWPer	Total length following all root surfaces	cm	111.7–1061.7	539.1	510.2 ± 163.8**
Network surface area	NWSA	The sum of surface area of all roots in the network	cm^2^	7.03–53.93	26.79	26.49 ± 7.94**
Network volume	NWV	The total volume of all roots in the network	cm^3^	0.08–0.48	0.25	0.252 ± 0.074*
Network width	NWW	The maximum linear width attained by the root	cm	4.17–21.33	12.42	12.43 ± 3.12**
Network width to depth	NWW_Dep	Ratio of NWW to NWDep (NWW/NWDep)	na	0.203–1.316	0.64	0.632 ± 0.159*
Root dry weight	RDWt	Oven dried root weight	g	0.008–0.045	0.02	0.024 ± 0.006
Shoot dry weight	SDWt	Oven dried shoot weight	g	0.015–0.064	0.03	0.028 ± 0.008*
Shoot length	SL	Length of shoot from root collar to tip of the shoot	cm	4.95–13.27	7.67	7.91 ± 1.30*
Shoot : Root ratio	S_RDWt	Ratio of SDWt to RDWt (SDWt/RDWt)	na	0.70–3.00	1.00	1.30 ± 0.44
Specific root length1	SRL	Ratio of NWL to NWV(NWL/NWV)	cm^-2^	686.4–1249	990.4	976.6 ± 116.4**
Specific root length2	SRL2	Ratio of NWL to RDWt (NWL/RDWt)	cm/g	5,615–19,685.5	10877	10,765.99 ± 2,695.79

^1^cm = centimeter; g = gram; na = not applicable.

^2^SD = standard deviation; * and ** = significant at P < 0.05 and 0.001, respectively.

Shoot dry weight, shoot length, root dry weight, shoot to root dry weight ratio, and the 14 root traits measured by the GIA software were analyzed. Basic statistics of the 16 traits were computed for 111 genotypes (**Table 1**). Four genotypes with a single replicate were not included in the analysis. A one-way analysis of variance (ANOVA) was performed for each trait using R. Pearson pairwise correlation coefficients between traits were calculated and summarized using R package sjPlot (Lüdecke, 2017). To have insight into the potential effect of geography of origin, variation due to root network length among geographic regions was illustrated in boxplot using R.

### Genotyping and Genetic Data Analysis

SNP data of the entire flax core collection (n = 407) was previously generated after resequencing each genotypes using Illumina HiSeq 2000 platform in 100 bp paired-end mode to at an average coverage of 17X. The alignment, SNP call, and quality control such as removal of SNPs in long terminal repeat region were performed as previously described (Sertse et al., 2019). The SNP data for the mini-core collection was extracted from this core collection dataset.

### Single Nucleotide Polymorphism Filtering and Preparation of Datasets

For this study, only SNPs with no missing data were used. Because the minor allele frequency (MAF) of an SNP could differ between the core and the mini-core collections, we used two datasets for GWAS. The first dataset (7K) included all SNPs with no missing data regardless of their MAF in the mini-core collection because these had already previously been included based on MAF > 5% criteria in the core collection. The second dataset was smaller (3K) because SNPs with MAF < 5% in the mini-core collection *per se* were removed. The two datasets were analyzed separately and results were compared.

### Genetic Structure and Variation Analysis

To estimate the possible number of ancestral populations (K), a cross-validation technique (Alexander and Lange, 2011) was applied. Analysis of ancestral proportion of each genotype (Pritchard et al., 2000) was performed for K values ranging from 2 to 20 using sparse non-negative matrix factorization (sNMF) (Frichot et al., 2014) of the R package LEA (Frichot and François 2015) with default parameters except that the number of runs was increased from 10 to 20. The K value producing the lowest cross-validation error was accepted as the number of ancestral populations. The same package was used to visualize the cross-validation and to generate the structure plot. Neighbor-joining (NJ) phylogenetic and principal component (PC) analyses were performed using TASSEL v 5.2 (Bradbury et al., 2007). Results of the two analyses were summarized using Tree of Life (iTOL) v3 (Letunic and Bork, 2016) and R, respectively. Genotypes were assigned to the suggested ancestral populations based on their Q-matrix. Populations were named based on the passport data of their members indicating geography of origin.

### Phenotype-Genotype Association Analyses and Mapping

The use of multi-locus methods that capture small effect loci in complex polygenic traits such as in plant roots and shoots has recently become a feasible approach. To benefit the algorithmic merits of different models and support results of one by an other, it is also advantageous to apply multiple methods (Zhang et al., 2019). To assess the genetic variants underlying each root and shoot traits in this study, the multi-locus GWAS methods FASTmrEMMA (Wen et al., 2016), FASTmrMLM (Tamba and Zhang, 2018), ISIS EM-BLASSO (Tamba et al., 2017), mrMLM (Wang et al., 2016), pKWmEB (Ren et al., 2018), and pLARmEB (Zhang et al., 2017) included in the R package multi-locus random-SNP-effect mixed linear model (mrMLM) (Wen et al., 2017) were applied. The single locus genome scan method latent factor mixed linear model (LFMM) in the R package lfmm (Frichot et al., 2013) was also used. To control the type I error in multiple comparison, false discovery rate (FDR) correction at = 0.05 was applied (Benjamini and Hochberg, 1995) for all models to identify significant QTNs. For LFMM, Bonferonni correction factor at α = 0.05 (0.05/n, *n = the number of total SNPs*) was also used as a comparative methods. Quantitative trait loci (QTL) regions spanning 100 Kb up and downstream of all associated QTNs were examined for the predicted coding genes they harbored using the flax reference genome (You et al., 2018). Predicted functions of genes identified within each QTL were investigated based on their *Arabidopsis* orthologues (www.arabidopsis.org). Strongly associated SNPs and their putative underlying genes were illustrated on the flax pseudomolecules (You et al., 2018) using MapChart 2.3 (Voorrips, 2002). The favorability of alleles at QTNs detected by at least two of the multi-locus models with high phenotypic variance explained (PVE) (*R* *^2^* > 5%), was illustrated using box plot based on mean phenotypic value of genotypes with each allele.

For genes previously identified to encode for interacting proteins, protein interaction networks were constructed using the tool STRING V11 (https://string-db.org) (Szklarczyk et al., 2018). The interaction networks were constructed based on protein matchings searches in flax (*Linum usitatissimum*) with a minimum regulatory confidence of 0.95.

## Results

### Phenotypic Variation

From the two shoot and 14 root targeted traits, significant variation (*P* < 0.05) among genotypes was detected for all except for root dry weight, network depth, and network distribution (**Table 1**). In multiple comparison, genotype TMP-2530 (U_MAR_C_CN98193) was significantly outperformed at least one genotype for 10 of the 13 traits (**Supplementary Table 1**). TMP-2530 was also the only genotype that had significantly higher shoot dry weight and, it displayed a distinctive heavy root network (**Figure 1C**) compared to other genotypes such as Hanley for example (**Figure 1D**).

**Figure 2:**
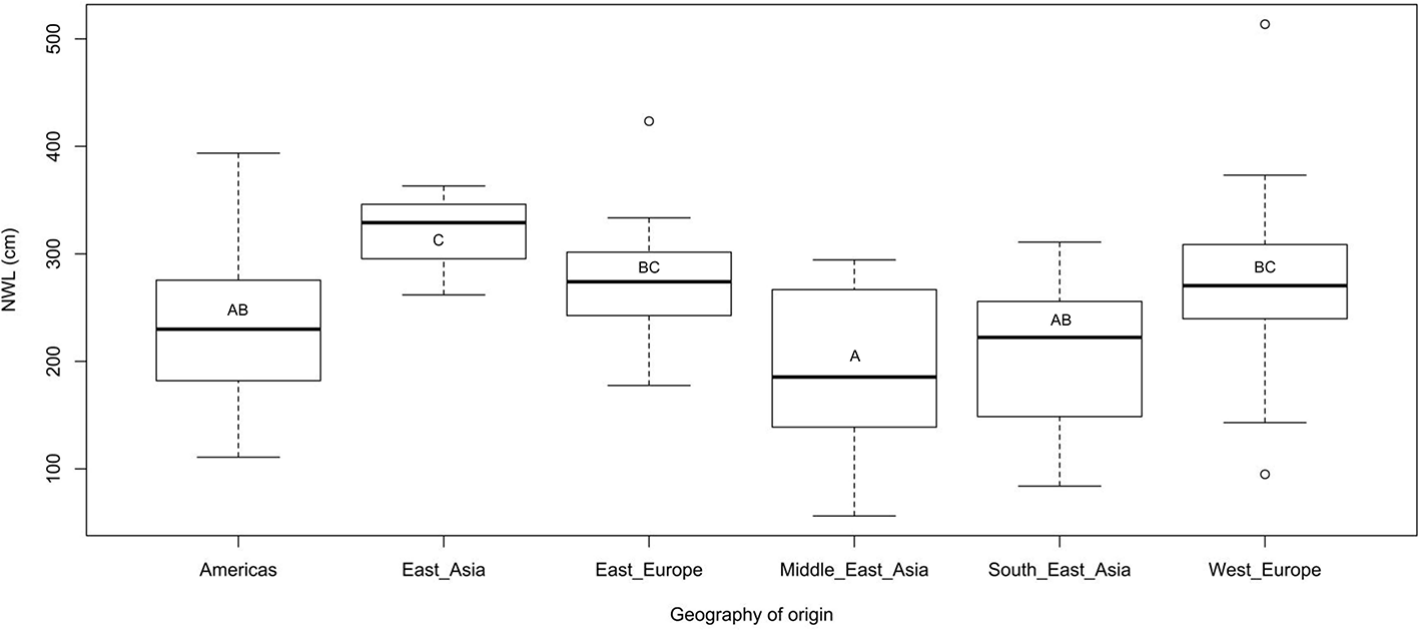
Box plot showing variation in root network length among genotypes based on their geography or origin. Letter/s in the box indicate (*P* < 0.05) after pairwise comparison.

Several traits were significantly correlated (**Table 2**). Shoot length and dry weight were strongly correlated with root dry weight and root network volume; the latter being highly correlated with root and shoot dry weights with r values of 0.78 and 0.58, respectively. East Asian genotypes appeared superior in network length (NWL) genotypes from Americas, the Middle-East, and South east Asia (**Figure 2**).

**Table 2:** Pairwise correlation of the traits.

Traits	ARD	MaxR	MedR	NWA	NWDep	NWDis	NWL	NWPer	NWSA	NWV	NWW	NWW_Dep	RDWt	SDWt	SL	S_RDWt	SRL1
**MaxR**	–0.52^***^																
**MedR**	–0.48^***^	0.87^***^															
**NWA**	–0.37^***^	0.75^***^	0.68^***^														
**NWDep**	–0.07	0.06	–0.11	0.37^***^													
**NWDis**	0.25^*^	–0.30^**^	–0.45^***^	–0.11	0.32^**^												
**NWL**	–0.48^***^	0.80^***^	0.72^***^	0.98^***^	0.34^***^	–0.16											
**NWPer**	–0.51^***^	0.84^***^	0.75^***^	0.98^***^	0.34^***^	–0.16	0.99^***^										
**NWSA**	–0.37^***^	0.75^***^	0.67^***^	0.99^***^	0.36^***^	–0.12	0.98^***^	0.97^***^									
**NWV**	–0.22^*^	0.64^***^	0.59^***^	0.97^***^	0.38^***^	–0.08	0.93^***^	0.91^***^	0.98^***^								
**NWW**	–0.36^***^	0.63^***^	0.53^***^	0.82^***^	0.34^***^	0.05	0.82^***^	0.82^***^	0.81^***^	0.77^***^							
**NWW_Dep**	–0.35^***^	0.62^***^	0.62^***^	0.65^***^	–0.17	–0.15	0.67^***^	0.66^***^	0.65^***^	0.59^***^	0.85^***^						
**RDWt**	–0.17	0.48^***^	0.43^***^	0.76^***^	0.33^***^	0.12	0.72^***^	0.71^***^	0.76^***^	0.76^***^	0.61^***^	0.45^***^					
**SDWt**	–0.08	0.44^***^	0.43^***^	0.62^***^	0.14	0.05	0.60^***^	0.59^***^	0.63^***^	0.63^***^	0.56^***^	0.50^***^	0.56^***^				
**SL**	–0.14	0.270^**^	0.17	0.41^***^	0.33^***^	0.24^*^	0.39^***^	0.40^***^	0.40^***^	0.39^***^	0.39^***^	0.19	0.45^***^	0.50^***^			
**S_RDWt**	0.16	–0.21^*^	–0.16	–0.35^***^	–0.35^***^	–0.04	–0.33^***^	–0.33^***^	–0.34^***^	–0.33^***^	–0.29^**^	–0.10	–0.63^***^	0.21^*^	–0.14		
**SRL1**	–0.80^***^	0.54^***^	0.51^***^	0.34^***^	0.13	–0.24^*^	0.47^***^	0.50^***^	0.32^***^	0.14	0.38^***^	0.33^***^	0.12	0.03	0.15	–0.21^*^	
**SRL2**	–0.4^***^	0.39^***^	0.35^***^	0.27^**^	0.02	–0.33^***^	0.35^***^	0.35^***^	0.28^**^	0.21^*^	0.26^**^	0.29^**^	–0.37^***^	0.05	–0.08	0.44^***^	0.454^***^

*P < 0.05; **P < 0.01,***P < 0.001.

### Single Nucleotide Polymorphisms and Genetic Structure

**Figure 3:**
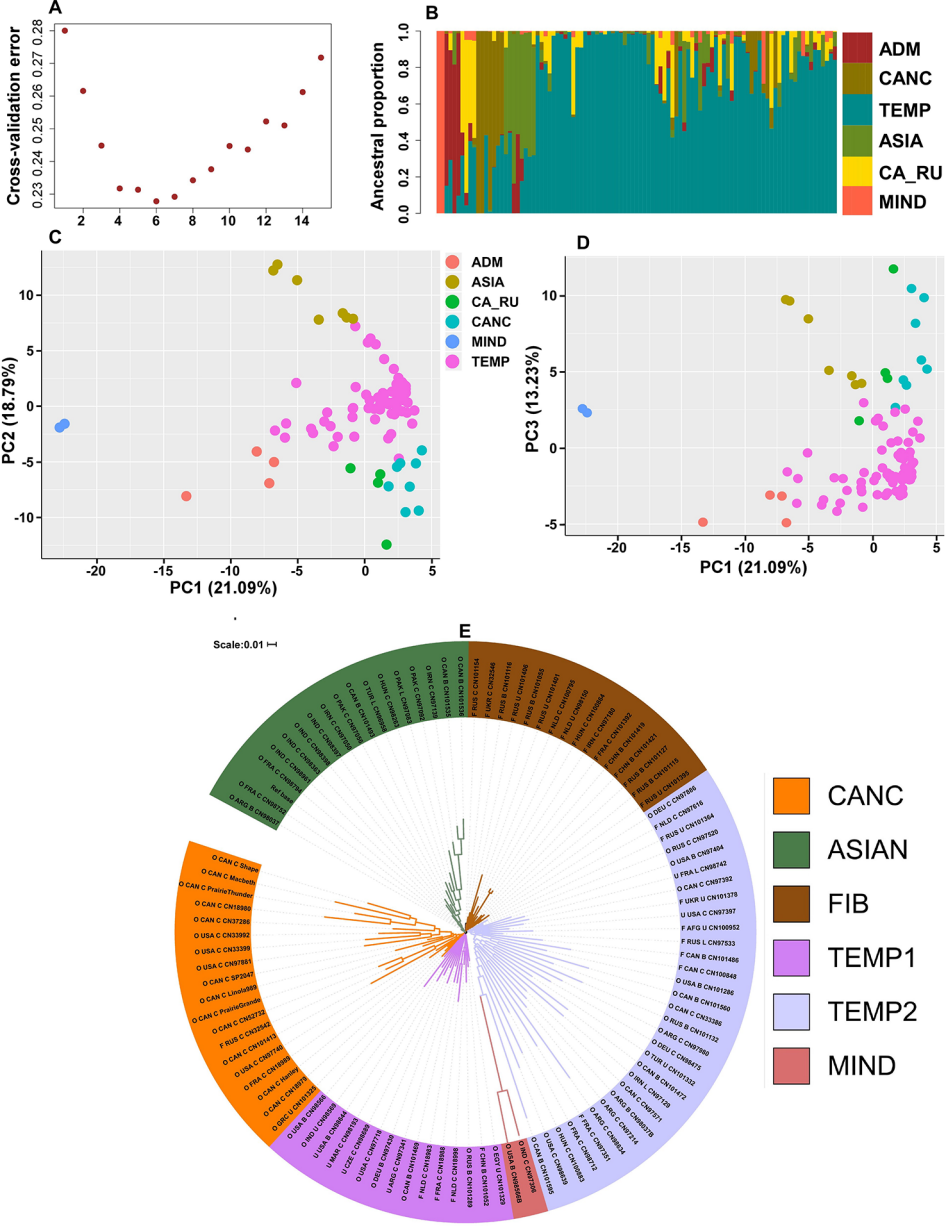
Population structure. **(A)** Estimate of the number of ancestral populations indicating six as the best fit, **(B)** population structure plot showing the six populations in different colors, **(C)** principal component analysis (PCA) plot of the first two principal components (PCs) where the percentages in parentheses represent the variance explained by the PCs, **(D)** PCA plot of the first and third PCs, **(E)** neighbor-joining (NJ) dendrogram where naming convention indicates the type (O, oil; F, fiber; U, unknown), the country of origin, the breeding status (C, cultivar; B, breeding material; L, landrace) followed by the accession name.

A total of 7707 SNPs with MAF > 5% in the core collection had no missing data and, 3,243 of these had a MAF > 5% in the mini-core *per se*. These datasets are henceforth referred to as the 7K and 3K datasets, respectively. Phenotypic data was incomplete for 4 genotypes and additional 10 genotypes had poor SNP call; hence the following analyses were carried out on 101 accessions of the mini-core collection using the 7K and 3K datasets independently. Population structure analysis based on both SNP datasets clustered the genotypes into six ancestral populations (**Figure 3A**) grouped as follows: Canadian cultivars (CANC), Canadian Russian (CA_RU), temperate (TEMP), Asian (ASIA), Admixture (ADM), and mini_Indian (MIND) with only two accessions (**Figure 3B**). Principal component analysis (PCAs) with PC1/PC2 and PC1/PC3 produced similar population structure patterns (**Figures 3C, D**). The NJ clustering slightly deviated from this pattern by assigning more than half of the fiber type accessions to a clade (FIB), splitting the TEMP into two clades (TEMP1 and TEMP2), distributing the CAN_RU, ADM, and MIND into the TEMP clades but with distinguishable high branch lengths of MIND. CANC and ASIA were each in a separate clade for a total of five clades and six distinguishable populations (**Figure 3E**).

### Quantitative Trait Nucleotide-Trait Association

**Figure 4:**
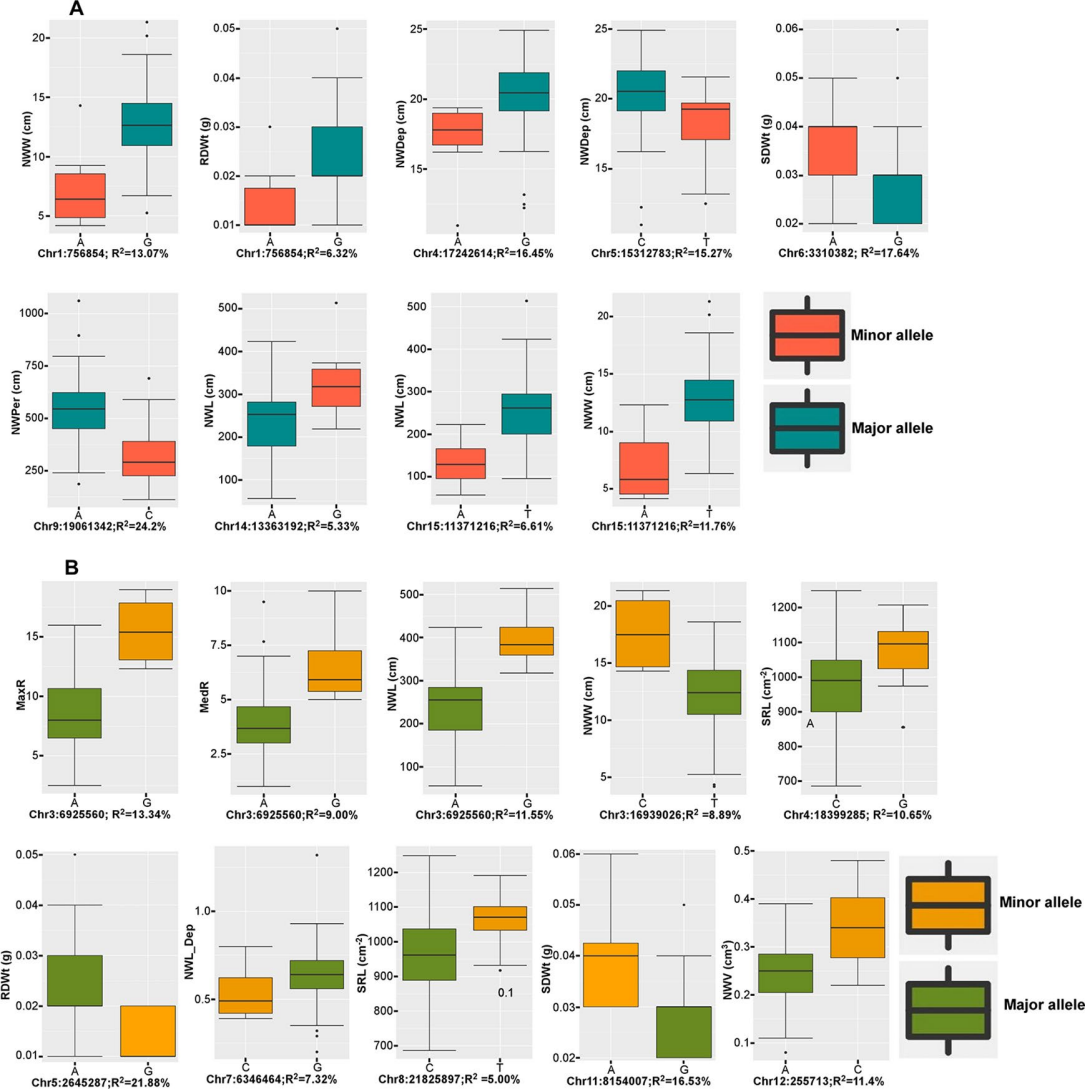
Box plots of large effect quantitative trait nucleotides (QTNs). **(A)** Phenotypic variations at large effect QTNs consistently detected in both 3k and 7k datasets, and **(B)** phenotypic variation at large effect QTNs that were detected by at least two models in at one or both datasets. Trait abbreviations are listed in **Table 1**.

From the two datasets, the six multi-locus (mrMLMs) and the LFMM methods identified a grand total of 228 QTNs associated with at least one of the 16 traits, of which 33 QTNs were detected in both datasets (**Supplementary Table 2**). A total of 35 large effect QTNs (high PVE) with *R* *^2^* > 5% for at least one trait were discovered, of which, 15 were identified by two or more models (**Table 3**). Overall, 14 QTNs were significantly associated with at least one trait with the LFMM model and the stringent Bonferroni 0.05/n threshold (**Supplementary Table 3**). The extent of the phenotypic variations for the traits at these 15 QTNs is illustrated (**Figures 4A, B**). Large effect QTNs at Chr4:17242614 and Chr5:15312783 positions were consistently associated with root network depth explaining 22.7 and 19.3% of variation for this trait respectively. Both were detected by at least four of the six multi-locus models and the LFMM model where the latter was also significant in the 3K dataset using the Bonferroni correction threshold (**Supplementary Table 3**, **Supplementary Figure 1**). A QTN associated with network perimeter with the highest PVE (*R* *^2^* = 24.20) at Chr9:19061342 using the multilocus FASTmrMLM (**Table 3**, **Supplementary Table 2**) was also significantly associated with the same trait based the Bonferroni criterion in both dataset (**Supplementary Table 3**, **Supplementary Figures 1** and **2**).

**Table 3:** QTNs with high phenotypic variance explained (R^2^ > 5%).

Traits	Model^1^	QTN^2^	R^2^ (%)^3^	LOD^4^	MAF^5^
NWW; NWW_Dep; RDWt	3,6; 3; 5	Chr1:756854*	6.32–13.07	3.1–6.0	6.06
RDWt	5	Chr1:4908649	8.50	4.8	7.07
MedR	4	Chr1:11064283	7.82	3.9	7.07
SDWt	4	Chr1:18970469	10.65	3.6	8.08
SDWt	5	Chr1:20356976	8.04	6.7	6.06
NWDis	3	Chr2:4513304	11.49	3.4	5.94
NWW_Dep	2	Chr2:5963452	14.88	3.9	9.9
NWDis	4	Chr2:7095057	10.54	3.4	10.1
MaxR; MedR; NWL; NWPer; NWA; NWSA	2; 3; 3,6; 6; 3 6	Chr3:6925560	9.00–13.34	3.7–4.4	3.96
NWW	3,6	Chr3:16939026	8.89	4.2–4.6	3.96
SDWt	4	Chr3:17343476	7.48	3.7	8.08
NWDis	3	Chr3:18772054	9.68	4.206	4.95
NWDis	4	Chr3:25380098	12.72	3.7	13.13
NWDep	2,3,4,6	Chr4:17242614*	7.18–22.68	3.6–5.1	7.07
SRL	2,3,5,6	Chr4:18399285	10.65–15.60	3.3–4.9	10.1
MedR	4	Chr5:1375386	12.94	4.0	9.09
RDWt	3,7	Chr5:2645287	21.88	4.2	6.93
MedR	4	Chr5:11019409	10.32	3.4	6.06
NWDep; NWL; NWSA	1,2,3,4,6; 6; 4	Chr5:15312783*	5.61–19.26	3.0–7.4	15.15
SDWt	5,6	Chr6:3310382*	11.45–17.64	3.3	13.13
SRL	3	Chr6:7732273	5.47	3.2	9.9
MedR	4	Chr7:4774423	7.72	3.025	6.06
NWW_Dep	3,4	Chr7:6346464	7.32–13.46	3.7–3.9	10.1
SRL	3,6	Chr8:21825897	5.00	3.4	13.86
RDWt	5	Chr9:15946848	9.16	5.3	7.07
NWPer	3,7	Chr9:19061342*	24.20	4.0–9.8	11.88
MedR	2	Chr11:5382629	8.27	3.2	5.94
SDWt	3,7	Chr11:8154007	16.53	4.8	7.92
NWV	3,6	Chr12:255713	11.34–11.40	3.1–3.2	5.94
SL	3	Chr12:3690290	12.58	3.3	5.94
SDWt	4	Chr12:12200657	10.05	3.5	18.18
NWL; NWPer; NWA; NWSA	4,6; 4; 4; 4	Chr14:13363192*	5.33–10.69	3.2–4.6	9.09
NWW_Dep	3	Chr14:15462441	14.77	5.0	2.97
MedR	4	Chr15:10531332	8.52	3.8	6.06
NWL; NWPer; NWA; NWSA; NWV; NWW	3,4,6; 3,4,6 3,4,6; 2,3,4,6 2,3,6; 2,4	Chr15:11371216*	5.36–14.17	3.0–4.6	6.06

^1^Models are 1 = FASTmrEMMA, 2 = FASTmrMLM, 3 = ISIS EM-BLASSO, 4 = mrMLM, 5 = pKWmEB, 6 = pLARmEB; LFMM = 7 (only QTN that passed the Bonferroni correction criterion).

^2^QTN, quantitative trait nucleotide, chromosome number and position are indicated, asterisks (*) indicate QTNs detected by multiple models from both 7K and 3K datasets.

^3^R2, coefficient of determination explaining phenotypic variation due to allelic effect.

^4^LOD, logarithm of odds.

^5^MAF, minor allele frequency.

**Figure 5:**
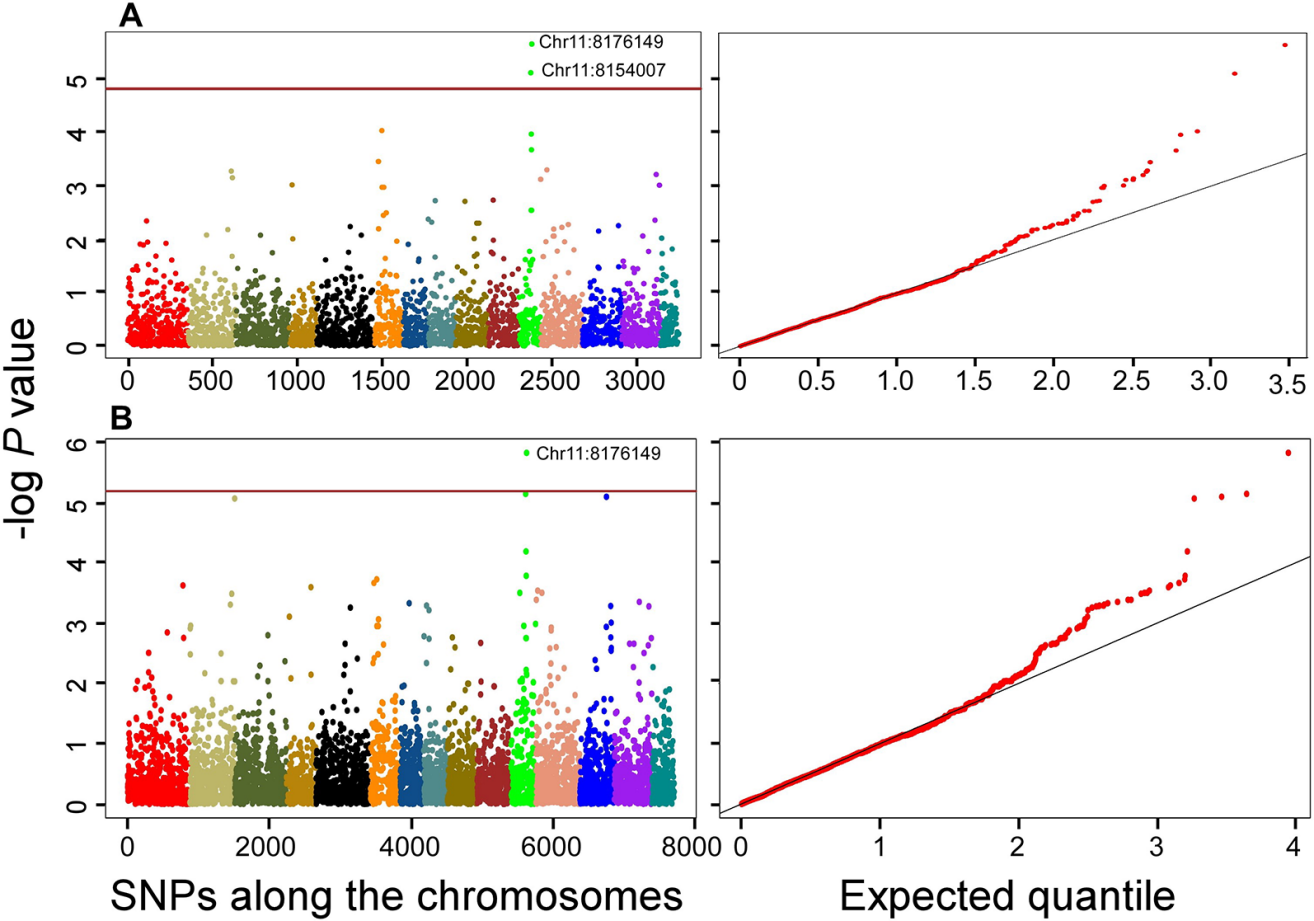
Manhattan and quantile-quantile plots showing QTNs that are significantly associated with shoot dry weight using the stringent Bonferroni criterion for both datasets; **(A)** 3K dataset; **(B)** 7K data set. The horizontal brown lines indicate the threshold *P* = 0.05/n = 0.05/3243 = 1.54178E-05 and 0.05/7707 = 6.48761E-06 for the 3K and 7K datasets, respectively. Position of the significant QTNs on chromosome 11 are indicated. Colors in Manhattan plot indicate the 15 chromosomes of flax in order from 1 to 15.

As expected, QTN associations with multiple correlated traits were observed. For example, a QTN at Chr15:11371216 position was associated with the following six correlated root network traits: network area, NWL, network perimeter, network surface area, network volume, and network width (**Supplementary Table 3**). This QTN was one of the large effect QTNs detected by multiple models for all the traits in both datasets (**Table 2** and **Supplementary Table 2**). Two QTNs (Chr6:3310382 and Chr11:8154007) were associated with shoot dry weight (SDWt), each explaining more than 15% of the phenotypic variance of the trait (**Table 3**). QTN Chr11:8154007 was also significant for SDWt in both datasets using the Bonferroni correction criterion (**Figure 5**).

### Genes Linked to Quantitative Trait Nucleotides

**Figure 6:**
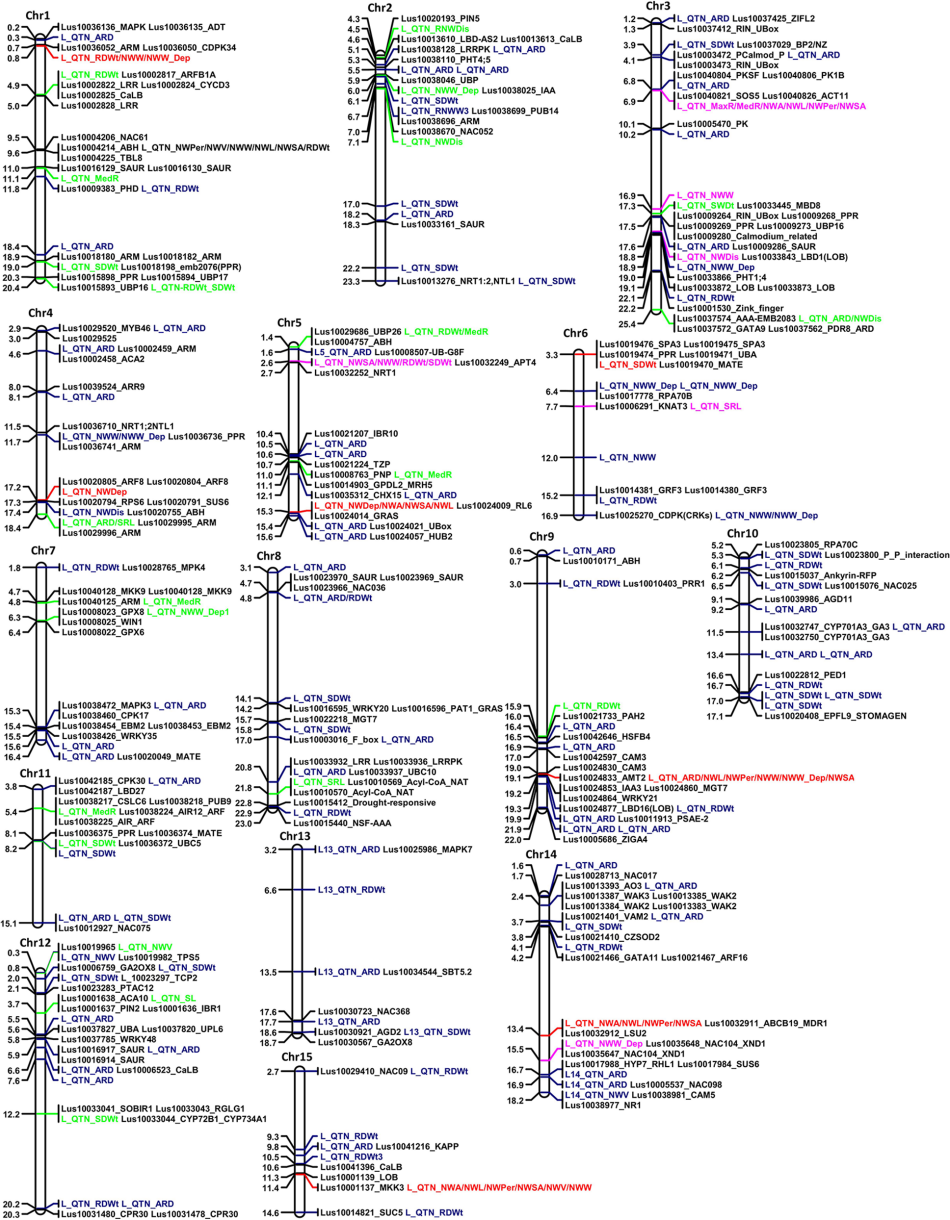
Physical map of the 15 chromosomes of flax illustrating the position of the quantitative trait nucleotides (QTNs) and their proximal candidate genes (right of the chromosomes). QTNs with R^2^ > 5% in the 3K, 7K or in both datasets are in green, purple, and red, respectively. QTNs indicated in blue have R^2^ < 5% and were detected in the 3K dataset. Numbers on the left of the chromosomes represent physical distances in megabases.

Most loci within 100 kb up and downstream of the detected QTNs harbored genes that had previously been reported to play role(s) in root and/or shoot development in plants (**Figure 6**, **Supplementary Table 2**). The genes were primarily related to auxin efflux, nutrient transport, and plant immunity. Loci corresponding to large effect QTNs detected by multiple methods harbored genes for the growth and development of plant organs. For instance, the locus defined by QTN Chr15:11371216 harbored genes predicted to encode a lateral organ boundary (LOB) protein and a mitogen-activated protein kinase (MAPK). Network analysis indicated that this predicted MAPK gene likely interacts with other MAPK genes typical of MAPK cascades (**Supplementary Figure 3A**). QTN locus Chr5:15312783, also detected by multiple methods but in this case for its association with root depth, comprised genes predicted to encode GRAS [collective name for gibberellic acid insensitive (GAI), repressor of GA1 (RGA), and Scarecrow (SCR)] transcription factors (Gao et al., 2004). This locus also harbored genes that were predicted to encode ARM repeats, a GATA-type zinc finger transcription factor family protein and YUCCA6 (YUC6). The other large effected QTN associated with root depth (Chr4:17242614) was linked to *Arabidopsis* orthologue genes AT5G37020 and AT5G10360 that encode auxin response factor-8 (ARF8) and sucrose synthase-6 (SUS6), respectively.

Most QTNs associated with SDWt were at loci containing genes predicted to encode pentatricopeptide repeat (PPR), photomorphogenesis, ubiquitin-related and plant defense proteins such as multi and toxic compound extrusion (MATE) (**Figure 6**, **Supplementary Table 2**). The large effect QTNs Chr6:3310382 and Chr11:8154007 associated with SDWt were located in relatively high gene density regions involved in plant immunity, development processes, and plant growth regulation (**Figure 6**, **Supplementary Table 2**). Among others, a gene predicted to function as a suppressor of phytochrome A-105 (SPA3) and assumed to regulate plant growth by suppressing photomorphogenesis (Laubinger and Hoecker 2003), was duplicated at QTN locus Chr6:3310382. The SPA3 and UBA proteins (**Figure 6**, **Supplementary Table 2**) were predicted to interact *via* COP1 (**Supplementary Figure 3B**).

## Discussion

Root trait measurements provide essential information to facilitate varietal improvement in breeding programs to select superior genotypes especially in nutrient and moisture deficit areas (Comas et al., 2013; Ndour et al., 2017). Root traits have already been used in breeding schemes to select elite genotypes in crops such as wheat for example (Wasson et al., 2014). Applications of root phenotype-genotype association through GWAS has enabled the identification of important QTL for root traits (Hochholdinger et al., 2018) that impact shoot traits including yield (Reinert et al., 2016). Our study provides insights into phenotype-genotype associations for early root and shoot traits of flax genotypes from over 20 countries by identifying QTL and proposing plausible candidate genes for further investigations.

### Phenotypic Variation

The significant variations observed among genotypes for most of the early root system and shoot development traits evaluated points to the genetic diversity of flax for such traits and the potential for genetic improvement. The highest and lowest morphometric values reflect the level of diversity within the gene pool, promising useful materials for improvement through breeding. The wide range of variation for most agronomic traits among flax genotypes (Richards and Diederichsen 2003) is well represented in the core collection (Diederichsen et al., 2013; You et al., 2017). Most genotypes in the flax core collection exhibit both fiber and oilseed features that can attributable to selection processes for dually elite ones (You et al., 2017).

Correlations between root and shoot traits reflect the notion of balance between roots and shoots referring to plants partitioning their resource allocations between the two plant parts (Davidson 1969; Garnier, 1991). A high correlation between shoot and root dry weights has also been reported in rice (Zhao et al., 2019); this is not surprising considering the role of roots in supplying nutrients to the above-ground parts. The higher correlations between root network traits such as network perimeter, network length, network surface area, and network volume with shoot traits compared to that with root depth suggest the crop's reliance on the top layer rooting system for resource uptake (Hocking et al., 1997; Kar et al., 2007).

The inverse relationship between average root diameter and root network related traits such as total network length, perimeter, and surface area have been reported not only in flax (Soto-Cerda et al., 2019) but also in other plants such as *Arabidopsis thaliana* (Qian et al., 2015). The strong negative correlation between specific root length and average root diameter agrees with similar trade-offs between these traits in plants such as maize (Zhu and Lynch, 2004) and various tree species (Bauhus and Messier, 1999; Kramer‐Walter et al., 2016). The superiority of East-Asian genotypes in root network length is consistent with the performance of these materials under drought condition (data not shown) that may reflect their breeding importance.

### Genome Wide Association and Candidate Genes

The genetic structure of the mini-core collection used herein is in accordance with previous reports describing the extent of the genetic and agronomic trait diversity of the flax core collection and indicating its suitability for GWAS (Soto-Cerda et al., 2013). Most QTN loci harbored genes predicted to play a role in organ development. QTN Chr15:11371216 was identified based on its association with multiple root traits, its large effect, and its detection by multiple models. The LOB and MAPK orthologous genes at this locus have already been shown to contribute to root development in *Medicago trunculata* (Liu et al., 2005; Ariel et al., 2010; Han et al., 2014). LOBs are plant-specific proteins known for their involvement in lateral organ development (Shuai et al., 2002) including lateral root formation (Jeon et al., 2017). LOB proteins not only mediate a number of root and shoot development processes but can also respond to environmental stimuli (Shuai et al., 2002; Xu et al., 2016). MAPKs regulate several physiological processes of all eukaryote organisms including microbes and metazoans (Nishihama et al., 1995). In plants, they are involved in diverse developmental processes including response to abiotic stresses (Nakagami et al., 2005) but they have also been reported to play a role in regulating plant root growth *via* auxin signaling (Zhao et al., 2013).

The consistent association of Chr5:15312783 with root depth based on most of the models used and the occurrence of multiple genes predicted to function as GRAS, ARM, ATA, and YUC genes at its locus hint at a possible role in determining root depth. GRAS family genes play important roles in plant organ development (Bolle, 2016). The predicted GRAS gene at this locus appeared to be an orthologue of *A. thaliana's* AT5G66770 that encodes SCR (Gao et al., 2004), thereby supporting the candidacy of this gene for the QTL. SCR and SHORTROOT (SHR) proteins of the GRAS family are known for their regulatory functions of root development in *Arabidopsis* (Benfey et al., 1993; Kamiya et al., 2003; Sbabou et al., 2010). These two proteins interact and work in tandem, i.e., SCR regulates the movement of SHR (Cui et al., 2007). *Arabidopsis* orthologues ARM (Coates et al., 2006), GATA (Behringer and Schwechheimer, 2015), and YUC (Woo et al., 2007; Cha et al., 2015) at this locus could also be important for the development of plant organs in general and roots in particular. The specific YUC6 orthologue gene at this locus was reported to have important role in drought tolerance in *Arabidopsis* (Cha et al., 2015). The large effect Chr4:17242614 QTN for root depth may be attributable to an ARF8 orthologous gene that can regulate auxin-mediated process and influence primary root elongation (Blilou et al., 2005) and possibly lateral roots as well (Wang et al., 2015; Lee et al., 2019). ARF8 has been demonstrated to have clear effect on root growth habit where the roots of wild type plants grew slanted and those of mutants had a vertical downward elongation (Tian et al., 2004). This gene can also regulate flower maturation in later developmental stage, affecting fertility and seed production (Nagpal et al., 2005; Ghelli et al., 2018). The existence of a SUS orthologous gene as a regulator of primary root development (Sturm et al., 1995) may also be entertained as a candidate QTL for flax root depth.

The consistent occurrence of PPR, MATE, and ubiquitin related orthologous genes at large effect QTN loci associated with shoot traits, especially with SDWt, make them strong candidates. The PPR genes are involved in plant growth and stress tolerance in several plant species (Laluk et al., 2011; Wu et al., 2016; Xing et al., 2018). MATE efflux proteins on the other hand play a vital role in plant immunity against different toxins (Diener et al., 2001) including secondary metabolites (Gomez et al., 2009), xenobiotics such as heavy metals (Li et al., 2002) and aluminum (Li et al., 2017). Ubiquitin orthologues linked to SDWt QTNs are candidate loci on the ground of their known roles in shoot development (Stirnberg et al., 2002; Yang et al., 2007). The SPA3 orthologous gene at the Chr6:3310382 locus, the QTN associated with SDWt, is a member of a gene family that acts as a suppressor of phytochrome A and regulates photomorphogenesis (Hoecker et al., 1999; Laubinger and Hoecker, 2003). In *Arabidopsis*, SPA3 has a pronounced effect on seedling elongation (Laubinger and Hoecker, 2003) and, interestingly, it is expressed in all above ground tissues while showing no detectable expression in roots (Zhu et al., 2008). The SPA3 protein in *Arabidopsis* and rice has a conserved DWD motif that reflects its role in modulating a protein involved in photomorphogenesis repression and in activation of etiolation through CONSTITUTIVELY PHOTOMORPHOGENIC1 (COP1); the latter also possesses E3 ubiquitin ligase activity (Lee et al., 2008). A secondary role for SPA3 that would be related to UBA at this locus through COP1 can therefore be considered (**Supplementary Figure 3**).

Most genes linked to the large effect QTNs associated with root and shoot traits are responsive to abiotic stresses such as drought, salt, and temperature. For instance, some GRAS family genes have useful roles in drought and salt tolerance (Ma et al., 2010; Xu et al., 2015). Some members of MAPK genes positively regulate low-temperature tolerance while decreasing drought and salt resistance (Jia et al., 2016). The role played by auxin-related genes (e.g., LOBs) in various abiotic stresses, including drought, have been reported for many plant species (Jain and Khurana, 2009; Wang et al., 2010; Jung et al., 2015; Huang et al., 2016). The QTN trait associations in these studies may further implicate variation in local adaptation to different environmental conditions given that our germplasm represented all flax growing regions of the world and represented more than 95% of the genetic diversity of whole flax core collection.

## Conclusions

Early root and shoot trait phenotyping and GWAS of flax have provided insights into the complex relationships of these traits and their associated QTN loci were mined to hypothesize candidate genes. Early root network traits are interrelated, positively impact shoot traits and, consequently, seedling vigor. As such, early root establishment may also affect downstream yield performance. Our results suggest that, in flax, the extent of the root network is more important than root depth *per se* during the early growth stages. Root development studies spanning all growth stages would be necessary to quantify the relative importance of both traits over the entire cropping season and under different moisture regimes.

The GWAS yielded QTNs associated with most of the root traits and both shoot traits and the candidate genes identified at the major loci provide grounds for further investigations, particularly as they relate to stress tolerance. Some of the QTNs have pleiotropic effects that can either stem from linked genetic features at the loci or from single genes affecting multiple root traits. SDWt associated loci harbored genes that regulates physiological processes in above ground plant parts such as photosynthesis. Genes expressed at different stages and in different tissues may be tested for their specific role in controlling agronomic traits or imparting stress tolerance. Therefore, some of these loci co-locate with QTL for other traits not measured herein. Given the polygenic nature of several agronomic traits, consideration must be give to QTL of small effects because their cumulative impact is important in pre-breeding and positive selection can be achieve through genomic selection and other marker-assisted breeding schemes.

## Data Availability Statement

This manuscript contains previously unpublished data. The name of the repository and accession number(s) are not available.

## Author Contributions

DS and SC conceived the project. DS, SC, and SR participated in designing and implementing the phenotyping experiment. SC and FY made the genetic data available. DS performed the phenotypic and GWAS data analysis. DS prepared the manuscript. SC, FY, and SR worked on revision of the manuscript.

## Funding

This Agri-Innovation Project (J-000279) was funded by the Flax Council of Canada and Agriculture and Agri-Food Canada.

## Conflict of Interest

The authors declare that the research was conducted in the absence of any commercial or financial relationships that could be construed as a potential conflict of interest.
