# Supplementary figures and images for: The Complex Genetic Architecture of Early Root and Shoot Traits in Flax Revealed by Genome-Wide Association Analyses
Source: Front Plant Sci. 2019 Nov 19;10:1483. doi: 10.3389/fpls.2019.01483 (PMC6878218; doi:10.3389/fpls.2019.01483)

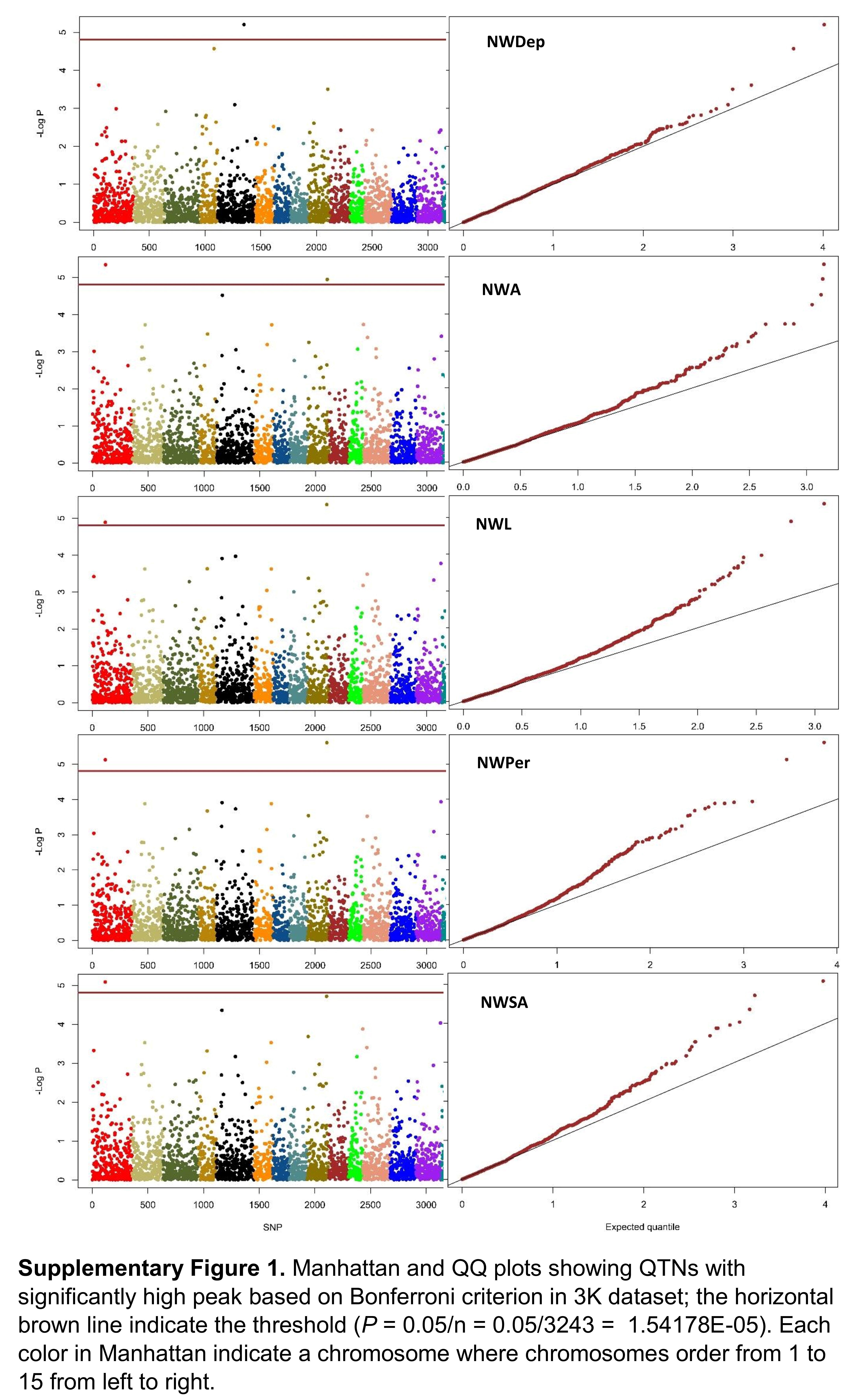

Supplement: Supplementary file 1 [file Image_1.jpeg]

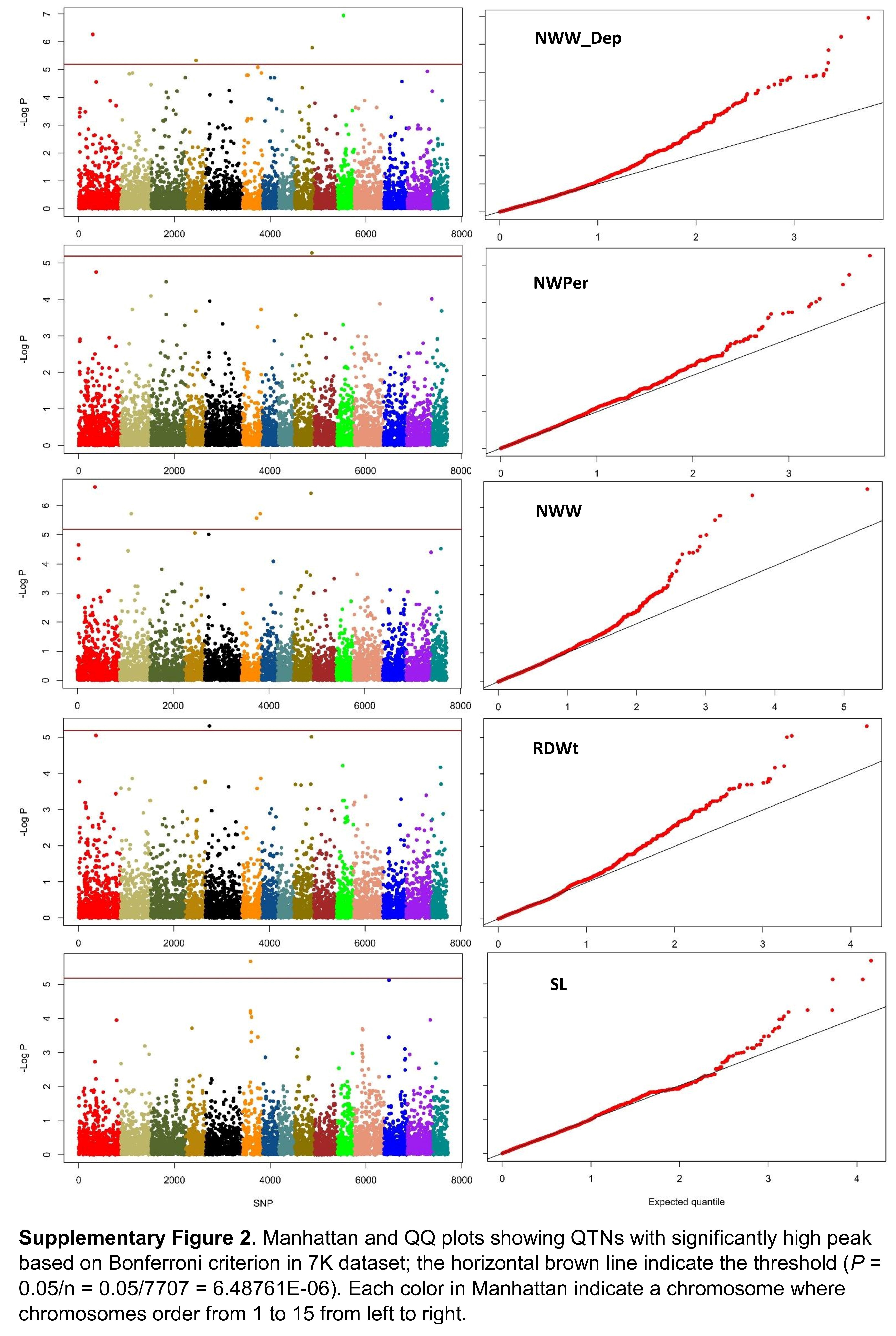

Supplement: Supplementary file 2 [file Image_2.jpeg]

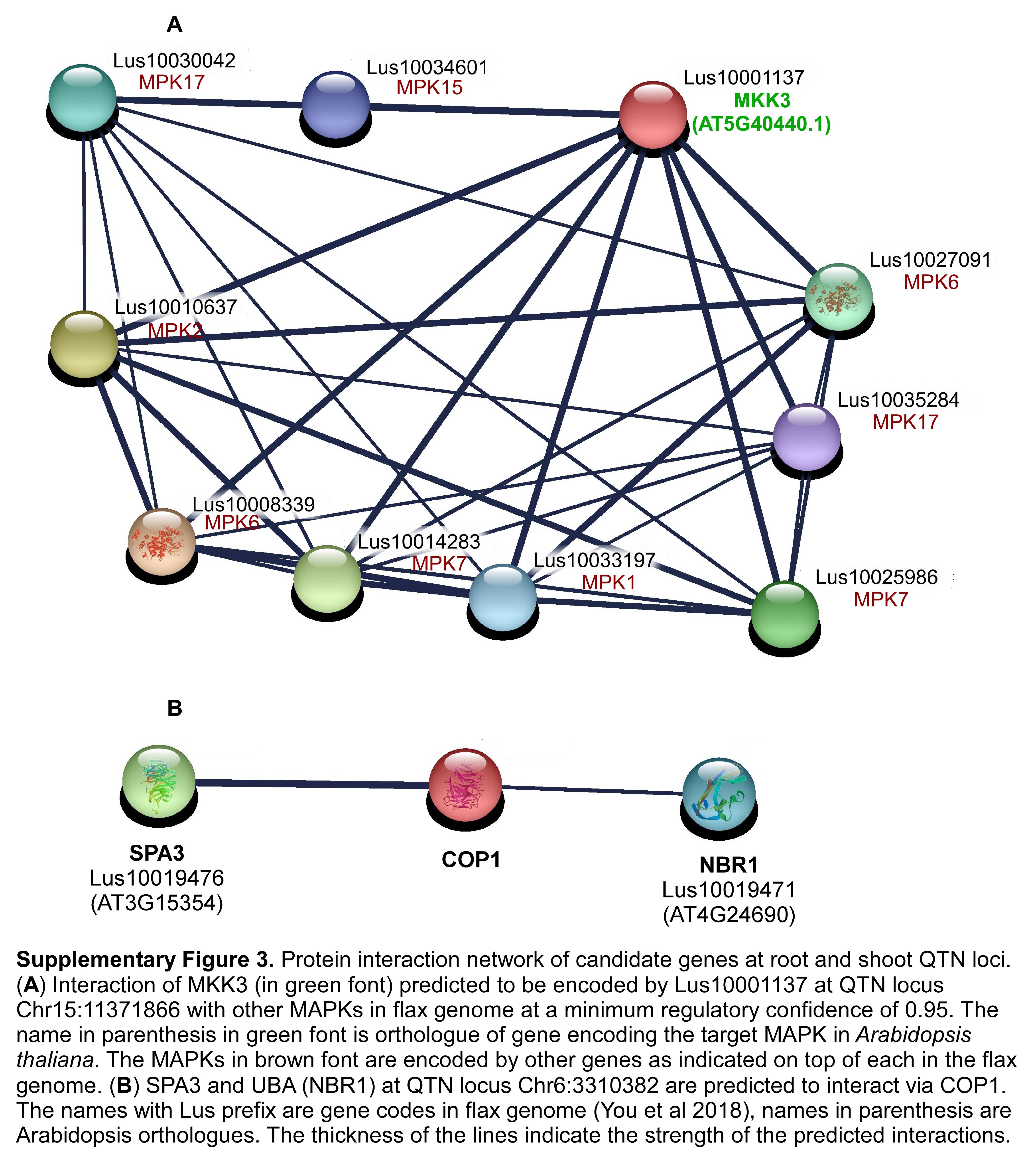

Supplement: Supplementary file 3 [file Image_3.jpeg]
