# Supplementary material for: The Complex Genetic Architecture of Early Root and Shoot Traits in Flax Revealed by Genome-Wide Association Analyses
Source: Front Plant Sci. 2019 Nov 19;10:1483. doi: 10.3389/fpls.2019.01483 (PMC6878218; doi:10.3389/fpls.2019.01483)
Supplement: Supplementary file 4 [file Table_1.docx]

**Supplementary Table 1.** List of superior genotypes for each traits that showed significant variation in ANOVA

| Trait^1^ | Population Mean±SD^2^ | Superior genotype | | Genotype Mean±SD^5^ | Superiority  over^6^ |
| --- | --- | --- | --- | --- | --- |
|  |  | TMP name^3^ | Passport name^4^ |  |  |
| MaxR | 8.94±3.37 | TMP-2530 | U_MAR_C_CN98193 | 19±7.07 | 16 |
|  |  | TMP-8216-5 | U_CZE_C_CN98689 | 17.5±3.54 | 7 |
|  |  | TMP-1922 | F_CHN_B_CN101419 | 15.67±2.31 | 5 |
| MedR | 3.99±1.65 | TMP-2530 | U_MAR_C_CN98193 | 10±4.24 | 15 |
|  |  | TMP-7590 | O_RUS_C_CN97520 | 9.5±4.95 | 9 |
| NWPer | 510.2±163.8 | TMP-2530 | U_MAR_C_CN98193 | 1061.73±170.8 | 8 |
|  |  | TMP-1155 | F_FRA_C_CN18986 | 820.83±212.21 | 1 |
|  |  | TMP-10020 | O_CAN_B_CN101595 | 796.39±253.18 | 1 |
| NWA | 7.27±2.15 | TMP-2530 | U_MAR_C_CN98193 | 14.67±2.52 | 4 |
|  |  | TMP-1155 | F_FRA_C_CN18986 | 11.86±0.61 | 1 |
| NWL | 245.3±77.5 | TMP-2530 | U_MAR_C_CN98193 | 513.66±73.18 | 7 |
|  |  | TMP-10020 | O_CAN_B_CN101595 | 393.71±144.81 | 1 |
|  |  | TMP-1155 | F_FRA_C_CN18986 | 391.42±52.7 | 1 |
| NWSA | 26.49±7.94 | TMP-2530 | U_MAR_C_CN98193 | 53.93±9.26 | 5 |
|  |  | TMP-1155 | F_FRA_C_CN18986 | 43.62±2.41 | 1 |
| NWV | 0.252±0.074 | TMP-2530 | U_MAR_C_CN98193 | 0.48±0.1 | 1 |
| SDWt | 0.028±0.008 | TMP-2530 | U_MAR_C_CN98193 | 0.062±0.022 | 8 |
| SL | 7.91±1.30 | TMP-8152-12 | U_ARG_C_CN97341 | 13.27±3.1 | 9 |
| SRL | 976.6±116.4 | TMP-1833 | F_RUS_C_CN101094 | 1240.50±159.89 | 5 |
|  |  | TMP-1855 | F_RUS_B_CN101116 | 1207.78±82.5 | 3 |
| ARD | 0.035±0.0001 | TMP-8284 | O_FRA_C_CN98752 | 0.041±0.002 | 3 |
|  |  | TMP-8168-3 | U_USA_B_CN98644 | 0.04±0.003 | 3 |
|  |  | TMP-2650-14 | O_TUR_L_CN96958 | 0.04±0.001 | 2 |
| NWW_Depth | 0.632±0.159 | TMP-2530 | U_MAR_C_CN98193 | 1.32±0.001 | 9 |
| NWW | 12.43±3.12 | TMP-2530 | U_MAR_C_CN98193 | 21.33±0.31 | 1 |

^1^Abrevation of the traits (see Table 1 for details and units)

^2^Overall mean and standard deviation (SD)

^3^TMP=temporary followed by temporary accession number at plant gene resource of Canada (PGRC), names used during experiment.

^4^Name in passport; naming convention indicates the type (O=oil type, F=fiber type, U= unknown), the originating country, the breeding status of the genotype (C=cultivar; B=breeding material; L=landrace) and accession number at PGRC.

^5^Mean and SD of the superior genotype

^6^Number of genotype over which the superior genotype has significantly higher value.
