# Supplementary material for: The Complex Genetic Architecture of Early Root and Shoot Traits in Flax Revealed by Genome-Wide Association Analyses
Source: Front Plant Sci. 2019 Nov 19;10:1483. doi: 10.3389/fpls.2019.01483 (PMC6878218; doi:10.3389/fpls.2019.01483)
Supplement: Supplementary file 5 [file Table_2.docx]

**Supplementary Table 2.** QTN detected in both 3 and 7K datasets using seven models

| **Trait** | **QTN^1^** | **Model** | **Dataset^2^** | **LOD^3^** | **R^2^ (%)^4^** | **MAF^5^** | ***P* value** | **Predicted gene** | **At orthologue^6^** | **Gene product** |
| --- | --- | --- | --- | --- | --- | --- | --- | --- | --- | --- |
| ARD | Chr1:250405 | pLARmEB | 3 | 23.2 | 0.57 | 21.78 | Inf | Lus10036136 | AT3G45640 | MAPK3 |
|  |  |  |  |  |  |  |  | Lus10036135 | AT3G07630 | ADT2 |
| NWW | Chr1:513047 | LFMM | 7 |  |  |  | 2.22E-05 |  |  |  |
| RDWt | Chr1:741582 | pLARmEB | 7 | 10.5 | 0.05 | 7.92 | 3.82E-12 | Lus10036052 | AT2G26780 | ARM |
| RDWt | Chr1:756854 | pKWmEB | 3 | 3.1 | 6.32 | 6.06 | 1.59E-04 | Lus10036050 | AT5G19360 | CPK34 |
| NWW | Chr1:756854 | ISIS EM-BLASSO | 7 | 6.0 | 13.07 | 5.94 | 1.55E-07 | Lus10036049 | AT1G62350 | PPR |
| NWW_Dep | Chr1:756854 | ISIS EM-BLASSO | 7 | 4.7 | 11.04 | 5.94 | 3.27E-06 |  |  |  |
| NWW | Chr1:756854 | LFMM | 7 |  |  |  | 6.65E-05 |  |  |  |
| NWW | Chr1:756854 | pLARmEB | 7 | 3.1 | 13.07 | 5.94 | 1.43E-04 |  |  |  |
| RDWt | Chr1:1122450 | pLARmEB | 7 | 4.8 | 0.00 | 1.98 | 2.42E-06 | Lus10035975 | AT2G17270 | PHT3;3 |
| RDWt | Chr1:3031443 | pLARmEB | 7 | 18.3 | 0.02 | 1.98 | Inf | Lus10042414 | AT4G31820 | NPY1 |
| RDWt | Chr1:4908649 | pKWmEB | 3 | 4.8 | 8.50 | 7.07 | 2.39E-06 | Lus10002819 | AT5G02910 | RIN_FB |
|  |  |  |  |  |  |  |  | Lus10002820 | AT3G58930 | RIN_FB |
|  |  |  |  |  |  |  |  | Lus10002822 | AT2G15320 | LRR |
|  |  |  |  |  |  |  |  | Lus10002825 | AT4G34150 | CaLB |
|  |  |  |  |  |  |  |  | Lus10002828 | AT3G19320 | LRR |
| NWW_Dep | Chr1:7339896 | LFMM | 7 |  |  |  | 5.39E-07 | Lus10029174 | AT3G53710 | AGD6 |
| NWL | Chr1:9613871 | LFMM | 3 |  |  |  | 1.29E-05 | Lus10004206 | AT3G44350 | NAC061 |
| NWPer | Chr1:9613871 | LFMM | 3 |  |  |  | 7.43E-06 | Lus10004214 | AT3G11620 | ABH |
| NWSA | Chr1:9613871 | LFMM | 3 |  |  |  | 8.16E-06 | Lus10004225 | AT3G11570 | TBL8 |
| NWV | Chr1:9613871 | LFMM | 3 |  |  |  | 8.57E-06 |  |  |  |
| NWW | Chr1:9613871 | LFMM | 3 |  |  |  | 1.52E-05 |  |  |  |
| RDWt | Chr1:9613871 | LFMM | 3 |  |  |  | 2.90E-05 |  |  |  |
| NWSA | Chr1:9613871 | LFMM | 7 |  |  |  | 1.46E-05 |  |  |  |
| NWA | Chr1:9613871 | LFMM | 7 |  |  |  | 6.94E-06 |  |  |  |
| NWW | Chr1:9613871 | LFMM | 7 |  |  |  | 2.29E-07 |  |  |  |
| RDWt | Chr1:9613871 | LFMM | 7 |  |  |  | 8.91E-06 |  |  |  |
| MedR | Chr1:11064283 | mrMLM | 3 | 3.9 | 7.82 | 7.07 | 2.09E-05 | Lus10016129/30 | AT2G28085 | SAUR |
| RDWt | Chr1:11839052 | pKWmEB | 3 | 3.5 | 3.66 | 29.29 | 5.29E-05 | Lus10009383 | AT5G22760 | PHD |
| RDWt | Chr1:12047870 | pLARmEB | 7 | 12.0 | 0.01 | 1.98 | 1.12E-13 | Lus10009398 | AT2G29940 | PDR3 |
| RDWt | Chr1:18212245 | pKWmEB | 3 | 4.0 | 0.36 | 9.09 | 1.77E-05 |  |  |  |
| ARD | Chr1:18434560 | pLARmEB | 3 | 10.5 | 0.33 | 12.87 | 3.48E-12 |  |  |  |
| ARD | Chr1:18434560 | ISIS EM-BLASSO | 7 | 3.7 | 0.06 | 12.87 | 3.49E-05 |  |  |  |
| SDWt | Chr1:18858986 | FASTmrMLM | 3 | 4.3 | 0.01 | 6.93 | 7.65E-06 |  |  |  |
| SDWt | Chr1:18970469 | mrMLM | 3 | 3.2 | 10.65 | 8.08 | 1.37E-04 | Lus10018180 | AT1G77600 | ARM |
| SDWt | Chr1:18970469 | FASTmrMLM | 7 | 6.0 | 0.00 | 7.92 | 1.46E-07 |  |  |  |
| RDWt | Chr1:19577662 | pLARmEB | 7 | 4.0 | 0.07 | 7.92 | 1.93E-05 | Lus10020829 | AT2G01420 | PIN4 |
| SDWt | Chr1:20356976 | pKWmEB | 3 | 6.7 | 8.04 | 6.06 | 2.60E-08 | Lus10015898 | AT2G13600 | PPR |
| SDWt | Chr1:20356976 | FASTmrMLM | 3 | 19.8 | 1.74 | 5.94 | Inf | Lus10015894 | AT5G65450 | UBP17 |
| RDWt | Chr1:20356976 | FASTmrMLM | 3 | 4.5 | 0.44 | 5.94 | 5.41E-06 | Lus10015893 | AT4G24560 | UBP16 |
| RDWt | Chr1:20356976 | ISIS EM-BLASSO | 3 | 3.4 | 0.39 | 5.94 | 8.16E-05 |  |  |  |
| SDWt | Chr1:20356976 | FASTmrMLM | 7 | 22.8 | 0.62 | 5.94 | Inf |  |  |  |
| RDWt | Chr1:20356976 | pLARmEB | 7 | 4.8 | 0.26 | 5.94 | 2.31E-06 |  |  |  |
| SDWt | Chr1:20356976 | pLARmEB | 7 | 3.2 | 0.00 | 5.94 | 1.13E-04 |  |  |  |
| ARD | Chr1:21006827 | pLARmEB | 3 | 23.4 | 0.03 | 9.9 | Inf |  |  |  |
| SDWt | Chr1:22813857 | FASTmrMLM | 7 | 3.4 | 0.01 | 6.93 | 7.26E-05 | Lus10014632 | AT2G14820 | NPY2 |
| SDWt | Chr1:25703627 | FASTmrMLM | 7 | 4.3 | 0.01 | 14.85 | 7.97E-06 | Lus10003106/7 | AT3G04290 | LTL1 |
| NWDis | Chr2:4513304 | ISIS EM-BLASSO | 3 | 3.4 | 11.49 | 5.94 | 7.22E-05 | Lus10020193 | AT5G16530 | PIN5 |
| NWDis | Chr2:4513304 | FASTmrMLM | 7 | 3.2 | 0.00 | 5.94 | 1.29E-04 | Lus10013610 | AT1G65620 | AS2 |
| ARD | Chr2:5139403 | pLARmEB | 3 | 4.9 | 0.09 | 6.93 | 2.16E-06 | Lus10038128 | AT5G20690 | LRR |
| ARD | Chr2:5452843 | pLARmEB | 3 | 4.4 | 0.17 | 17.82 | 7.16E-06 | Lus10038110 | AT5G20380 | PHT4;5 |
| ARD | Chr2:5529143 | pLARmEB | 3 | 27.0 | 0.06 | 5.94 | Inf |  |  |  |
| NWW | Chr2:5554181 | LFMM | 7 |  |  |  | 3.57E-05 |  |  |  |
| NWW | Chr2:5554185 | LFMM | 7 |  |  |  | 3.57E-05 |  |  |  |
| NWW_Dep | Chr2:5963452 | FASTmrMLM | 3 | 3.9 | 14.88 | 9.9 | 2.25E-05 | Lus10038046 | AT3G15580 | UBP |
| SDWt | Chr2:6056808 | FASTmrMLM | 3 | 10.4 | 0.17 | 5.94 | 4.80E-12 | Lus10038025 | AT3G62100 | IAA30 |
| NWW | Chr2:6662071 | LFMM | 3 |  |  |  | 2.92E-05 | Lus10038699 | AT3G54850 | PUB14 |
| NWW | Chr2:6662071 | LFMM | 7 |  |  |  | 1.88E-06 | Lus10038696 | AT1G01950 | ARK2 |
| NWDis | Chr2:7095057 | mrMLM | 3 | 3.4 | 10.54 | 10.1 | 7.81E-05 | Lus10038670 | AT3G10490 | NAC052 |
| SDWt | Chr2:16999042 | FASTmrMLM | 7 | 10.7 | 0.00 | 0.99 | 2.22E-12 |  |  |  |
| NWW | Chr2:18121207 | pLARmEB | 7 | 3.2 | 4.39 | 8.91 | 1.27E-04 | Lus10025840 | AT5G66870 | ASL1 |
| ARD | Chr2:18237395 | pLARmEB | 3 | 3.2 | 0.06 | 6.93 | 1.22E-04 | Lus10033161 | AT1G75590 | SAUR |
| RDWt | Chr2:21629463 | pLARmEB | 7 | 13.2 | 0.59 | 13.86 | 7.00E-15 |  |  |  |
| SDWt | Chr2:22207057 | FASTmrMLM | 3 | 3.0 | 0.02 | 9.9 | 2.01E-04 |  |  |  |
| SDWt | Chr2:22207057 | FASTmrMLM | 7 | 7.7 | 0.00 | 9.9 | 2.37E-09 |  |  |  |
| RDWt | Chr2:22305709 | pLARmEB | 7 | 6.2 | 0.09 | 10.89 | 8.44E-08 |  |  |  |
| SDWt | Chr2:23342970 | ISIS EM-BLASSO | 3 | 3.7 | 0.02 | 6.93 | 3.96E-05 | Lus10013276 | AT1G69850 | NRT1:2 |
| SDWt | Chr2:23342970 | FASTmrMLM | 7 | 10.9 | 0.03 | 6.93 | 1.35E-12 |  |  |  |
| SDWt | Chr2:25271724 | LFMM | 7 |  |  |  | 8.61E-06 |  |  |  |
| SDWt | Chr2:25271724 | pLARmEB | 7 | 4.6 | 0.01 | 2.97 | 3.99E-06 |  |  |  |
| ARD | Chr3:1231621 | pLARmEB | 3 | 6.9 | 0.01 | 5.94 | 1.60E-08 | Lus10037425 | AT3G43790 | ZIFL2 |
| SDWt | Chr3:1363801 | FASTmrMLM | 7 | 37.7 | 0.00 | 10.89 |  |  |  |  |
| SDWt | Chr3:2215745 | FASTmrMLM | 7 | 13.7 | 0.00 | 5.94 | 1.78E-15 |  |  |  |
| SDWt | Chr3:3857902 | FASTmrMLM | 3 | 3.1 | 0.00 | 5.94 | 1.54E-04 |  |  |  |
| ARD | Chr3:4092867 | pLARmEB | 3 | 5.5 | 0.45 | 17.82 | 4.52E-07 |  |  |  |
| SDWt | Chr3:4897266 | FASTmrMLM | 7 | 17.8 | 0.00 | 5.94 |  |  |  |  |
| RDWt | Chr3:5009686 | pLARmEB | 7 | 5.6 | 0.03 | 9.9 | 3.38E-07 |  |  |  |
| RDWt | Chr3:5432287 | pLARmEB | 7 | 7.8 | 0.08 | 12.87 | 2.06E-09 |  |  |  |
| ARD | Chr3:6835049 | pLARmEB | 3 | 18.1 | 0.04 | 7.92 |  |  |  |  |
| MaxR | Chr3:6925560 | FASTmrMLM | 7 | 3.8 | 13.34 | 3.96 | 3.04E-05 | Lus10040829 | AT2G28840 | XBAT31 |
| MedR | Chr3:6925560 | ISIS EM-BLASSO | 7 | 3.9 | 9.00 | 3.96 | 2.48E-05 | Lus10040826 | AT3G12110 | ACT11 |
| NWSA | Chr3:6925560 | ISIS EM-BLASSO | 7 | 4.0 | 10.04 | 3.96 | 1.67E-05 | Lus10040817/18/19/20 | AT5G07900 | MTERF |
| NWL | Chr3:6925560 | ISIS EM-BLASSO | 7 | 4.4 | 11.55 | 3.96 | 6.40E-06 |  |  |  |
| NWA | Chr3:6925560 | pLARmEB | 7 | 3.7 | 9.21 | 3.96 | 3.64E-05 |  |  |  |
| NWL | Chr3:6925560 | pLARmEB | 7 | 4.4 | 11.41 | 3.96 | 6.40E-06 |  |  |  |
| NWPer | Chr3:6925560 | pLARmEB | 7 | 4.1 | 10.31 | 3.96 | 1.33E-05 |  |  |  |
| SDWt | Chr3:7074112 | FASTmrMLM | 7 | 3.3 | 0.16 | 6.93 | 9.82E-05 |  |  |  |
| SDWt | Chr3:7668335 | FASTmrMLM | 7 | 5.8 | 0.09 | 4.95 | 2.22E-07 |  |  |  |
| ARD | Chr3:10151903 | pLARmEB | 3 | 4.5 | 0.14 | 5.94 | 5.57E-06 |  |  |  |
| NWW | Chr3:16939026 | ISIS EM-BLASSO | 7 | 4.6 | 8.89 | 3.96 | 4.21E-06 | Lus10033528 | AT4G39370 | UBP27 |
| NWW | Chr3:16939026 | pLARmEB | 7 | 4.2 | 8.89 | 3.96 | 1.16E-05 | Lus10033524 | AT2G40890 | CYP98A3 |
| SDWt | Chr3:17343476 | mrMLM | 3 | 3.7 | 7.48 | 8.08 | 3.68E-05 | Lus10033446 | AT4G08920 | CRY1, |
| ARD | Chr3:17343476 | pLARmEB | 3 | 18.2 | 0.06 | 9.9 | Inf | Lus10033447 | AT1G31885 | NIP3;1 |
| RDWt | Chr3:18363136 | pLARmEB | 7 | 11.4 | 0.02 | 4.95 | 4.00E-13 |  |  |  |
| NWDis | Chr3:18772054 | ISIS EM-BLASSO | 7 | 4.2 | 9.68 | 4.95 | 1.08E-05 |  |  |  |
| NWW_Dep | Chr3:18933706 | LFMM | 3 |  |  |  | 2.31E-05 |  |  |  |
| RDWt | Chr3:22145720 | pKWmEB | 3 | 5.2 | 0.82 | 7.07 | 9.16E-07 |  |  |  |
| ARD | Chr3:25380098 | FASTmrMLM | 3 | 8.7 | 0.05 | 12.87 | 2.21E-10 |  |  |  |
| ARD | Chr3:25380098 | FASTmrMLM | 7 | 9.6 | 0.11 | 12.87 | 3.30E-11 |  |  |  |
| NWDis | Chr3:25380098 | mrMLM | 3 | 3.7 | 12.72 | 13.13 | 3.90E-05 | Lus10037572 | AT4G32890 | GATA9 |
| ARD | Chr4:2910990 | pLARmEB | 3 | 26.9 | 0.18 | 10.89 | Inf | Lus10029520 | AT5G12870 | MYB46 |
|  |  |  |  |  |  |  |  | Lus10029525 | AT3G57670 | NTT,WIP2 |
| ARD | Chr4:4572761 | pLARmEB | 3 | 8.0 | 0.03 | 5.94 | 1.40E-09 | Lus10002459 | AT1G64960 | ARM |
|  |  |  |  |  |  |  |  | Lus10002458 | AT4G37640 | ACA2 |
| ARD | Chr4:8061253 | pLARmEB | 3 | 11.1 | 0.03 | 19.8 | 8.53E-13 | Lus10039524 | AT3G57040 | RR9,RR4 |
| RDWt | Chr4:8745388 | pLARmEB | 7 | 5.5 | 0.12 | 6.93 | 5.29E-07 |  |  |  |
| NWW_Dep | Chr4:11667975 | LFMM | 3 |  |  |  | 8.24E-05 | Lus10036736 | AT1G26900 | PPR |
| NWW | Chr4:11667975 | LFMM | 7 |  |  |  | 8.66E-06 | Lus10036742 | AT1G14300 | ARM |
| NWW_Dep | Chr4:11667975 | LFMM | 7 |  |  |  | 4.62E-06 |  |  |  |
| RDWt | Chr4:12310229 | pLARmEB | 7 | 4.8 | 0.06 | 5.94 | 2.59E-06 |  |  |  |
| SDWt | Chr4:17127643 | ISIS EM-BLASSO | 7 | 4.7 | 0.00 | 1.98 | 3.39E-06 |  |  |  |
| SDWt | Chr4:17127643 | pLARmEB | 7 | 4.0 | 0.00 | 1.98 | 1.82E-05 |  |  |  |
| NWDep | Chr4:17242614 | mrMLM | 3 | 5.1 | 22.68 | 7.07 | 1.17E-06 | Lus10020805 | AT5G37020 | ARF8 |
| NWDep | Chr4:17242614 | ISIS EM-BLASSO | 3 | 3.7 | 16.61 | 6.93 | 3.61E-05 | Lus10020804 | AT5G37020 | ARF8 |
| NWDep | Chr4:17242614 | FASTmrMLM | 3 | 4.3 | 16.61 | 6.93 | 9.16E-06 | Lus10020794 | AT5G10360 | RPS6B |
| NWDep | Chr4:17242614 | pLARmEB | 3 | 3.6 | 7.18 | 6.93 | 4.17E-05 | Lus10020791 | AT1G73370 | SUS6 |
| NWDep | Chr4:17242614 | LFMM | 3 |  |  |  | 2.69E-05 |  |  |  |
| NWDep | Chr4:17242614 | ISIS EM-BLASSO | 7 | 5.1 | 16.45 | 6.93 | 1.37E-06 |  |  |  |
| NWDis | Chr4:17408739 | FASTmrEMMA | 3 | 3.2 | 0.00 | 12.87 | 1.40E-04 | Lus10020755 | AT5G50890 | ABH |
| NWDis | Chr4:17408739 | FASTmrEMMA | 7 | 3.1 | 0.00 | 12.87 | 1.59E-04 |  |  |  |
| RDWt | Chr4:17943797 | pLARmEB | 7 | 8.5 | 0.08 | 4.95 | 3.89E-10 |  |  |  |
| SRL | Chr4:18399285 | pKWmEB | 3 | 3.3 | 15.60 | 10.1 | 9.60E-05 | Lus10029995 | AT2G05810 | ARM |
| SRL | Chr4:18399285 | FASTmrMLM | 3 | 4.8 | 11.05 | 9.9 | 2.91E-06 | Lus10029996 | AT2G05810 | ARM |
| SRL | Chr4:18399285 | pLARmEB | 3 | 4.9 | 10.65 | 9.9 | 2.16E-06 |  |  |  |
| SRL | Chr4:18399285 | ISIS EM-BLASSO | 3 | 4.9 | 10.65 | 9.9 | 2.16E-06 |  |  |  |
| ARD | Chr4:18399285 | pLARmEB | 3 | 19.4 | 2.27 | 9.9 | Inf |  |  |  |
| ARD | Chr4:18399285 | ISIS EM-BLASSO | 3 | 6.1 | 1.90 | 9.9 | 1.10E-07 | Lus10029686 | AT3G49600 | UBP26 |
| SDWt | Chr5:504766 | FASTmrMLM | 7 | 12.0 | 0.00 | 5.94 | 1.08E-13 |  |  |  |
| RDWt | Chr5:731588 | pLARmEB | 7 | 4.7 | 0.03 | 5.94 | 2.96E-06 |  |  |  |
| MedR | Chr5:1375386 | mrMLM | 3 | 4.0 | 12.94 | 9.09 | 1.76E-05 | Lus10004757 | AT5G47330 | ABH |
| RDWt | Chr5:1375386 | ISIS EM-BLASSO | 3 | 3.0 | 0.76 | 8.91 | 1.99E-04 |  |  |  |
| RDWt | Chr5:1375386 | FASTmrMLM | 3 | 5.3 | 0.39 | 8.91 | 7.16E-07 |  |  |  |
| ARD | Chr5:1610010 | pLARmEB | 3 | 13.2 | 0.01 | 10.89 | 6.66E-15 | Lus10008507 | AT4G16520 | G8F |
| NWSA | Chr5:2645287 | LFMM | 3 |  |  |  | 4.36E-05 | Lus10032249 | AT4G12440 | APT4 |
| RDWt | Chr5:2645287 | FASTmrMLM | 7 | 4.2 | 0.15 | 6.93 | 1.04E-05 | Lus10032252 | AT1G12110 | NRT1 |
| SDWt | Chr5:2645287 | FASTmrMLM | | 11.1 | 0.01 | 6.93 | 9.69E-13 |  |  |  |
| RDWt | Chr5:2645287 | ISIS EM-BLASSO | | 4.2 | 21.88 | 6.93 | 1.21E-05 |  |  |  |
| NWW | Chr5:2645287 | LFMM |  |  |  |  | 9.69E-06 |  |  |  |
| RDWt | Chr5:2645287 | LFMM |  |  |  |  | 4.83E-06 |  |  |  |
| RDWt | Chr5:9226031 | pLARmEB | 7 | 23.1 | 0.03 | 10.89 | Inf |  |  |  |
| SDWt | Chr5:9373438 | FASTmrMLM | 7 | 3.5 | 0.00 | 9.9 | 6.31E-05 |  |  |  |
| ARD | Chr5:10460806 | ISIS EM-BLASSO | 3 | 3.9 | 0.33 | 7.92 | 2.50E-05 |  |  |  |
| ARD | Chr5:10619678 | pLARmEB | 3 | 13.7 | 0.03 | 5.94 | 2.00E-15 | Lus10021224 | AT5G43630 | TZP |
| MedR | Chr5:11019409 | mrMLM | 3 | 3.4 | 10.32 | 6.06 | 7.46E-05 | Lus10008763 | AT5G14580 | PNP |
|  |  |  |  |  |  |  |  | Lus10014903 | AT4G26690 | GPDL2 |
| ARD | Chr5:12067166 | pLARmEB | 3 | 42.6 | 0.02 | 6.93 | Inf | Lus10035312 | AT2G13620 | CHX15 |
| NWDep | Chr5:15312783 | mrMLM | 3 | 5.6 | 19.26 | 15.15 | 4.03E-07 | Lus10028301 | AT3G50870 | GATA18 |
| NWDep | Chr5:15312783 | FASTmrMLM | 3 | 4.9 | 15.27 | 14.85 | 2.09E-06 | Lus10024009 | AT1G75250 | RL6,RSM3 |
| NWDep | Chr5:15312783 | ISIS EM-BLASSO | 3 | 4.2 | 15.27 | 14.85 | 1.20E-05 | Lus10024014 | AT5G66770 | GRAS |
| NWDep | Chr5:15312783 | FASTmrEMMA | 3 | 3.9 | 13.35 | 14.85 | 2.53E-05 | Lus10024022 | AT4G36650 | PBRP |
| NWSA | Chr5:15312783 | mrMLM | 3 | 3.1 | 11.48 | 15.15 | 1.67E-04 | Lus10024023 | AT5G25620 | YUC6 |
| NWDep | Chr5:15312783 | pLARmEB | 3 | 5.6 | 6.60 | 14.85 | 3.97E-07 |  |  |  |
| NWL | Chr5:15312783 | pLARmEB | 3 | 3.1 | 5.61 | 14.85 | 1.50E-04 |  |  |  |
| NWSA | Chr5:15312783 | pLARmEB | 3 | 3.1 | 4.97 | 14.85 | 1.67E-04 |  |  |  |
| NWA | Chr5:15312783 | pLARmEB | 3 | 3.1 | 4.64 | 14.85 | 1.70E-04 |  |  |  |
| NWDep | Chr5:15312783 | LFMM | 3 |  |  |  | 6.29E-06 |  |  |  |
| NWDep | Chr5:15312783 | FASTmrEMMA | 7 | 3.0 | 0.00 | 14.85 | 2.01E-04 |  |  |  |
| NWDep | Chr5:15312783 | ISIS EM-BLASSO | 7 | 5.7 | 10.41 | 14.85 | 2.82E-07 |  |  |  |
| NWDep | Chr5:15312783 | mrMLM | 7 | 3.0 | 15.48 | 15.15 | 2.01E-04 |  |  |  |
| RDWt | Chr5:15312783 | pLARmEB | 7 | 13.7 | 0.32 | 14.85 | 2.11E-15 |  |  |  |
| ARD | Chr5:15359894 | pLARmEB | 3 | 8.3 | 0.03 | 6.93 | 6.11E-10 |  |  |  |
| ARD | Chr5:15570504 | pLARmEB | 3 | 12.8 | 0.05 | 7.92 | 1.69E-14 | Lus10024057 | AT1G55250 | HUB2 |
| RDWt | Chr5:16359682 | pLARmEB | 7 | 3.9 | 0.56 | 12.87 | 2.34E-05 |  |  |  |
| SDWt | Chr6:3310382 | pKWmEB | 3 | 3.3 | 17.64 | 13.13 | 9.93E-05 | Lus10019475/6 | AT3G15354 | SAP3 |
| SDWt | Chr6:3310382 | FASTmrMLM | 7 | 6.6 | 0.35 | 12.87 | 3.44E-08 | Lus10019474 | AT3G48250 | PPR |
| SDWt | Chr6:3310382 | pLARmEB | 7 | 3.3 | 11.45 | 12.87 | 9.42E-05 | Lus10019471 | AT4G24690 | UBA/TS-N |
|  |  |  |  |  |  |  |  | Lus10019470 | AT5G52450 | MATE efflux |
| NWW_Dep | Chr6:6417516 | LFMM | 3 |  |  |  | 1.28E-05 | Lus10017778 | AT5G08020 | RPA70B |
| NWW_Dep | Chr6:6427626 | LFMM | 3 |  |  |  | 1.28E-05 |  |  |  |
| RDWt | Chr6:6698595 | pLARmEB | 7 | 36.4 | 0.03 | 3.96 | Inf |  |  |  |
| SDWt | Chr6:6704279 | FASTmrMLM | 7 | 8.4 | 0.00 | 1.98 | 5.06E-10 |  |  |  |
| RDWt | Chr6:6704279 | pLARmEB | 7 | 43.2 | 0.01 | 1.98 | Inf |  |  |  |
| SRL | Chr6:7732273 | ISIS EM-BLASSO | 7 | 3.2 | 5.47 | 9.9 | 1.37E-04 |  |  |  |
| NWW | Chr6:12030162 | LFMM | 3 |  |  |  | 5.78E-05 |  |  |  |
| NWW | Chr6:12030162 | LFMM | 7 |  |  |  | 1.88E-06 |  |  |  |
| RDWt | Chr6:13079727 | pLARmEB | 7 | 11.6 | 0.01 | 1.98 | 2.79E-13 |  |  |  |
| RDWt | Chr6:13140060 | pLARmEB | 7 | 3.9 | 0.03 | 3.96 | 2.51E-05 |  |  |  |
| SDWt | Chr6:14867203 | FASTmrMLM | 7 | 8.0 | 0.01 | 9.9 | 1.16E-09 |  |  |  |
| RDWt | Chr6:15202179 | pKWmEB | 3 | 4.4 | 1.81 | 8.08 | 6.70E-06 | Lus10014380/1 | AT2G36400 | GRF3 |
| RDWt | Chr6:15418446 | pLARmEB | 7 | 12.9 | 0.00 | 6.93 | 1.19E-14 |  |  |  |
| NWW | Chr6:16928785 | LFMM | 3 |  |  |  | 2.92E-05 | Lus10025270 | AT3G50530 | CRK |
| NWW_Dep | Chr6:16928785 | LFMM | 3 |  |  |  | 1.71E-04 |  |  |  |
| NWW | Chr6:16928785 | LFMM | 7 |  |  |  | 2.68E-06 |  |  |  |
| RDWt | Chr6:17011819 | pLARmEB | 7 | 6.9 | 0.01 | 10.89 | 1.80E-08 |  |  |  |
| RDWt | Chr7:1779542 | pKWmEB | 3 | 3.3 | 1.98 | 8.08 | 1.05E-04 | Lus10028765 | AT4G01370 | MPK4 |
| NWDis | Chr7:4694299 | LFMM | 7 |  |  |  | 8.56E-06 |  |  |  |
| NWDis | Chr7:4694715 | LFMM | 7 |  |  |  | 8.56E-06 |  |  |  |
| MedR | Chr7:4774423 | mrMLM | 3 | 3.0 | 7.72 | 6.06 | 1.90E-04 | Lus10040127/8 | AT1G73500 | MKK9 |
|  |  |  |  |  |  |  |  | Lus10040125 | AT3G02840 | ARM |
| RDWt | Chr7:6087128 | pLARmEB | 7 | 8.1 | 0.03 | 3.96 | 1.00E-09 |  |  |  |
| NWW_Dep | Chr7:6346464 | mrMLM | 3 | 3.9 | 13.45 | 10.1 | 2.27E-05 | Lus10008023 | AT1G63460 | GPX8 |
| NWW_Dep | Chr7:6346464 | ISIS EM-BLASSO | 3 | 3.7 | 7.32 | 9.9 | 3.87E-05 | Lus10008025 | AT1G80600 | WIN1 |
| NWW_Dep | Chr7:6346464 | FASTmrMLM | 3 | 3.3 | 0.00 | 9.9 | 1.00E-04 | Lus10008022 | AT4G11600 | GPX6 |
| NWW_Dep | Chr7:6346464 | FASTmrEMMA | 7 | 3.7 | 0.00 | 9.9 | 3.72E-05 |  |  |  |
| ARD | Chr7:6346464 | FASTmrMLM | 7 | 5.0 | 0.21 | 9.9 | 1.57E-06 |  |  |  |
| NWDep | Chr7:6759944 | ISIS EM-BLASSO | 7 | 4.6 | 0.00 | 2.97 | 4.06E-06 |  |  |  |
| ARD | Chr7:15286061 | pLARmEB | 3 | 11.9 | 0.03 | 5.94 | 1.42E-13 | Lus10038472 | AT3G45640 | MAPK3 |
|  |  |  |  |  |  |  |  | Lus10038460 | AT5G12180 | CPK17 |
| ARD | Chr7:15553408 | pLARmEB | 3 | 50.0 | 0.06 | 6.93 | Inf | Lus10038426 | AT2G34830 | WRKY35 |
| NWW | Chr7:16072649 | LFMM | 7 |  |  |  | 8.20E-05 |  |  |  |
| ARD | Chr7:16356085 | pLARmEB | 3 | 4.2 | 0.05 | 5.94 | 1.07E-05 | Lus10020049 | AT3G21690 | MATE efflux |
| ARD | Chr7:16356085 | FASTmrMLM | 7 | 4.5 | 0.04 | 5.94 | 4.82E-06 |  |  |  |
| RDWt | Chr7:17879110 | pLARmEB | 7 | 8.1 | 0.01 | 3.96 | 1.04E-09 |  |  |  |
| ARD | Chr8:3103449 | pLARmEB | 3 | 27.4 | 0.19 | 9.9 | Inf |  |  |  |
| RDWt | Chr8:4754353 | FASTmrMLM | 3 | 3.6 | 0.08 | 5.94 | 4.77E-05 | Lus10023970 | AT1G29450 | SAUR |
| ARD | Chr8:4754354 | pLARmEB |  | 25.0 | 0.00 | 5.94 | Inf | Lus10023966 | AT2G17040 | NAC036 |
| RDWt | Chr8:10360902 | pLARmEB | 7 | 18.6 | 0.00 | 2.97 | Inf |  |  |  |
| SDWt | Chr8:14140850 | FASTmrMLM | 3 | 8.6 | 0.09 | 7.92 | 3.38E-10 | Lus10016595 | AT4G26640 | WRKY20 |
|  |  |  |  |  |  |  |  | Lus10016596 | AT5G48150 | PAT1 |
| SDWt | Chr8:15837449 | FASTmrMLM | 3 | 3.5 | 0.11 | 8.91 | 6.12E-05 | Lus10022218 | AT5G09690 | MGT7 |
| ARD | Chr8:17030355 | pLARmEB | 3 | 18.7 | 0.03 | 5.94 | Inf | Lus10003016 | AT1G13570 | F_Box |
| RDWt | Chr8:17777940 | pLARmEB | 7 | 3.6 | 0.01 | 3.96 | 4.27E-05 |  |  |  |
| ARD | Chr8:20840576 | pLARmEB | 3 | 26.1 | 0.02 | 5.94 | Inf | Lus10033932 | AT3G20820 | LRR |
|  |  |  |  |  |  |  |  | Lus10033937 | AT5G53300 | UBC10 |
| SRL | Chr8:21825897 | pLARmEB | 3 | 3.4 | 5.00 | 13.86 | 7.84E-05 | Lus10010569 | AT3G22560 | AcylCoANAT |
| SRL | Chr8:21825897 | ISIS EM-BLASSO | 3 | 3.4 | 5.00 | 13.86 | 7.84E-05 | Lus10010570 | AT2G32030 | AcylCoANAT |
| RDWt | Chr8:22357078 | pLARmEB | 7 | 7.0 | 0.09 | 15.84 | 1.31E-08 |  |  |  |
| SDWt | Chr8:22898342 | FASTmrMLM | 7 | 8.8 | 0.00 | 3.96 | 1.85E-10 |  |  |  |
| RDWt | Chr8:22919544 | pKWmEB | 3 | 6.1 | 0.00 | 6.06 | 1.05E-07 | Lus10015412 | AT5G26990 | Drought |
| ARD | Chr9:630908 | pLARmEB | 3 | 26.4 | 0.23 | 9.9 |  | Lus10010171 | AT3G19970 | ABH |
| RDWt | Chr9:1259084 | pLARmEB | 7 | 9.5 | 0.05 | 5.94 | 3.54E-11 |  |  |  |
| RDWt | Chr9:2989474 | FASTmrMLM | 3 | 3.1 | 0.01 | 6.93 | 1.62E-04 | Lus10010403 | AT1G32100 | PRR1 |
| NWW_Dep | Chr9:4628185 | FASTmrMLM | 7 | 3.7 | 0.00 | 15.84 | 3.98E-05 |  |  |  |
| RDWt | Chr9:5639608 | pLARmEB | 7 | 5.6 | 0.12 | 9.9 | 3.94E-07 |  |  |  |
| RDWt | Chr9:15946848 | pKWmEB | 3 | 5.3 | 9.16 | 7.07 | 7.31E-07 | Lus10021733 | AT5G42870 | PAH2 |
| RDWt | Chr9:15946848 | pLARmEB | 3 | 4.0 | 0.99 | 6.93 | 1.66E-05 |  |  |  |
| SDWt | Chr9:15946848 | FASTmrMLM | 7 | 8.5 | 0.10 | 6.93 | 3.54E-10 |  |  |  |
| RDWt | Chr9:15946848 | pLARmEB | 7 | 66.2 | 0.87 | 6.93 | Inf |  |  |  |
| ARD | Chr9:16446802 | pLARmEB | 3 | 4.8 | 0.07 | 11.88 | 2.70E-06 | Lus10042646 | AT1G46264 | SCZ |
| ARD | Chr9:16933932 | pLARmEB | 3 | 9.7 | 0.24 | 8.91 | 2.30E-11 | Lus10042597 | AT3G56800 | CAM3 |
| RDWt | Chr9:18388080 | pLARmEB | 7 | 14.1 | 0.09 | 5.94 | 7.77E-16 |  |  |  |
| SDWt | Chr9:19039213 | FASTmrMLM | 7 | 6.5 | 0.00 | 4.95 | 4.20E-08 |  |  |  |
| RDWt | Chr9:19040305 | pLARmEB | 7 | 6.8 | 0.01 | 2.97 | 1.95E-08 |  |  |  |
| ARD | Chr9:19061342 | pLARmEB | 3 | 9.8 | 0.13 | 11.88 | 1.87E-11 | Lus10024830 | AT4G14640 | CAM8 |
| NWL | Chr9:19061342 | LFMM | 3 |  |  |  | 4.27E-06 | Lus10024833 | AT2G38290 | AMT2;1 |
| NWPer | Chr9:19061342 | LFMM | 3 |  |  |  | 2.43E-06 | Lus10024853 | AT1G04240 | IAA3 |
| NWSA | Chr9:19061342 | LFMM | 3 |  |  |  | 1.91E-05 |  |  |  |
| NWW | Chr9:19061342 | LFMM | 3 |  |  |  | 1.28E-05 |  |  |  |
| NWW_Dep | Chr9:19061342 | LFMM | 3 |  |  |  | 3.67E-05 |  |  |  |
| NWPer | Chr9:19061342 | FASTmrMLM | 7 | 4.0 | 24.20 | 11.88 | 1.98E-05 |  |  |  |
| NWSA | Chr9:19061342 | LFMM | 7 |  |  |  | 2.12E-05 |  |  |  |
| NWA | Chr9:19061342 | LFMM | 7 |  |  |  | 1.07E-05 |  |  |  |
| NWL | Chr9:19061342 | LFMM | 7 |  |  |  | 7.59E-06 |  |  |  |
| NWPer | Chr9:19061342 | LFMM | 7 |  |  |  | 5.24E-06 |  |  |  |
| NWW | Chr9:19061342 | LFMM | 7 |  |  |  | 3.76E-07 |  |  |  |
| NWW_Dep | Chr9:19061342 | LFMM | 7 |  |  |  | 1.62E-06 |  |  |  |
| RDWt | Chr9:19061342 | LFMM | 7 |  |  |  | 9.70E-06 |  |  |  |
| RDWt | Chr9:19295240 | FASTmrMLM | 3 | 5.3 | 0.24 | 5.94 | 8.59E-07 | Lus10024860 | AT5G09690 | MGT7 |
|  |  |  |  |  |  |  |  | Lus10024864 | AT2G30590 | WRKY21 |
|  |  |  |  |  |  |  |  | Lus10024877 | AT2G42430 | ASL18 |
| ARD | Chr9:19859864 | pLARmEB | 3 | 11.3 | 0.06 | 8.91 | 5.24E-13 | Lus10011913 | AT2G20260 | PSAE-2 |
| ARD | Chr9:21921657 | ISIS EM-BLASSO | 3 | 3.3 | 0.05 | 5.94 | 1.03E-04 | Lus10005686 | AT1G08680.4 | AGD14 |
| ARD | Chr9:21921657 | pLARmEB | 3 | 88.8 | 0.03 | 5.94 | Inf |  |  |  |
| ARD | Chr9:21925137 | pLARmEB | 3 | 4.7 | 0.03 | 5.94 | 3.00E-06 |  |  |  |
| RDWt | Chr10:5257573 | pKWmEB | 3 | 3.4 | 2.33 | 8.08 | 7.69E-05 | Lus10023805 | AT5G45400 | RPA70C |
| SDWt | Chr10:5257573 | FASTmrMLM | 7 | 6.3 | 0.02 | 7.92 | 6.90E-08 |  |  |  |
| RDWt | Chr10:5257573 | pLARmEB | 7 | 10.1 | 0.23 | 7.92 | 9.83E-12 |  |  |  |
| RDWt | Chr10:6106628 | pKWmEB | 3 | 4.0 | 2.92 | 7.07 | 1.82E-05 | Lus10015037 | AT3G09550 | Ankyrin |
| RDWt | Chr10:6247028 | pLARmEB | 7 | 8.1 | 0.92 | 11.88 | 1.12E-09 |  |  |  |
| SDWt | Chr10:6457226 | pKWmEB | 3 | 3.0 | 2.05 | 6.06 | 1.88E-04 | Lus10015076 | AT1G61110 | NAC025 |
| RDWt | Chr10:7751366 | pLARmEB | 7 | 5.7 | 0.04 | 3.96 | 3.15E-07 |  |  |  |
| ARD | Chr10:9207013 | pLARmEB | 3 | 7.0 | 0.18 | 9.9 | 1.38E-08 | Lus10039986 | AT3G07490 | AGD11 |
| ARD | Chr10:11511478 | pLARmEB | 3 | 12.1 | 0.01 | 8.91 | 8.44E-14 | Lus10032747 | AT5G25900 | CYP701A3 |
| ARD | Chr10:13371316 | pLARmEB | 3 | 22.7 | 0.12 | 9.9 | Inf |  |  |  |
| ARD | Chr10:13391033 | pLARmEB | 3 | 10.8 | 0.07 | 9.9 | 1.91E-12 |  |  |  |
| RDWt | Chr10:14815706 | pLARmEB | 7 | 5.0 | 0.01 | 2.97 | 1.61E-06 |  |  |  |
| ARD | Chr10:16154433 | ISIS EM-BLASSO | 7 | 4.8 | 0.14 | 4.95 | 2.45E-06 |  |  |  |
| RDWt | Chr10:16677317 | | 3 | 3.3 | 0.00 | 17.82 | 1.02E-04 | Lus10022812 | AT2G33150.1 | PKT3 |
| RDWt | Chr10:16677317 | | 7 | 42.4 | 0.84 | 17.82 | Inf |  |  |  |
| ARD | Chr10:16680000 | pLARmEB | 3 | 28.5 | 0.05 | 19.8 | Inf |  |  |  |
| ARD | Chr10:16956264 | pLARmEB | 3 | 25.5 | 0.53 | 16.83 | Inf |  |  |  |
| ARD | Chr10:16957410 | pLARmEB | 3 | 5.5 | 0.88 | 17.82 | 4.88E-07 |  |  |  |
| SDWt | Chr10:17003973 | FASTmrMLM | 3 | 8.3 | 0.02 | 5.94 | 6.00E-10 | Lus10020408 | AT4G12970 | EPFL9 |
| SDWt | Chr10:17003973 | FASTmrMLM | 7 | 32.7 | 0.01 | 5.94 | Inf |  |  |  |
| SDWt | Chr11:578136 | FASTmrMLM | 7 | 7.3 | 0.00 | 2.97 | 5.98E-09 |  |  |  |
| RDWt | Chr11:3194902 | pLARmEB | 7 | 14.8 | 0.02 | 4.95 | 1.11E-16 |  |  |  |
| SDWt | Chr11:3430032 | FASTmrMLM | 7 | 14.7 | 0.00 | 6.93 | 2.22E-16 |  |  |  |
| ARD | Chr11:3783939 | pLARmEB | 3 | 7.8 | 0.17 | 6.93 | 2.25E-09 | Lus10042185 | AT1G74740 | CPK30 |
|  |  |  |  |  |  |  |  | Lus10042187 | AT3G47870 | LBD27,SCP |
| NWW_Dep | Chr11:5123720 | LFMM | 7 |  |  |  | 1.13E-07 |  |  |  |
| MedR | Chr11:5382153 | FASTmrMLM | 3 | 3.2 | 8.27 | 5.94 | 1.15E-04 | Lus10038217 | AT3G07330 | CSLC6 |
|  |  |  |  |  |  |  |  | Lus10038218 | AT3G07360 | PUB9 |
|  |  |  |  |  |  |  |  | Lus10038224 | AT3G07390 | ARF-AIR12 |
|  |  |  |  |  |  |  |  | Lus10038225 | AT3G25290 | ARF |
| SDWt | Chr11:6948325 | FASTmrMLM | 7 | 5.5 | 0.00 | 7.92 | 4.93E-07 |  |  |  |
| SDWt | Chr11:8154007 | ISIS EM-BLASSO | 3 | 4.8 | 16.53 | 7.92 | 2.68E-06 | Lus10036375 | AT2G46050 | PPR |
| SDWt | Chr11:8154007 | LFMM | 3 |  |  |  | 7.74E-06 | Lus10036374 | AT4G00350 | MATE efflux |
| SDWt | Chr11:8154007 | FASTmrMLM | 7 | 3.5 | 0.86 | 7.92 | 5.82E-05 |  |  |  |
| SDWt | Chr11:8154007 | LFMM | 7 |  |  |  | 7.21E-06 |  |  |  |
| SDWt | Chr11:8176149 | LFMM | 3 |  |  |  | 2.24E-06 | Lus10036372 | AT1G63800 | UBC5 |
| SDWt | Chr11:8176149 | FASTmrMLM | 7 | 4.3 | 2.78 | 6.93 | 9.41E-06 |  |  |  |
| SDWt | Chr11:8176149 | LFMM | 7 |  |  |  | 1.50E-06 |  |  |  |
| RDWt | Chr11:14272770 | pLARmEB | 7 | 19.9 | 0.01 | 9.9 | Inf |  |  |  |
| ARD | Chr11:15115639 | pLARmEB | 3 | 34.0 | 0.02 | 7.92 | Inf |  |  |  |
| SDWt | Chr11:15133773 | FASTmrMLM | 3 | 9.2 | 0.00 | 6.93 | 7.15E-11 | Lus10012927 | AT4G29230 | NAC075 |
| NWV | Chr12:255713 | ISIS EM-BLASSO | 3 | 3.2 | 11.40 | 5.94 | 1.13E-04 | Lus10019965 | AT2G23810 | TET8 |
| NWV | Chr12:255713 | pLARmEB | 3 | 3.1 | 11.34 | 5.94 | 1.49E-04 | Lus10019982 | AT4G17770 | TPS5 |
| NWV | Chr12:255749 | LFMM | 3 |  |  |  | 4.70E-05 |  |  |  |
| SDWt | Chr12:348747 | FASTmrMLM | 7 | 3.6 | 0.00 | 2.97 | 4.21E-05 |  |  |  |
| RDWt | Chr12:348747 | pLARmEB | 7 | 31.2 | 0.06 | 2.97 | Inf |  |  |  |
| SDWt | Chr12:799432 | FASTmrMLM | 3 | 4.9 | 0.00 | 5.94 | 2.10E-06 | Lus10006759 | AT4G21200 | GA2OX8 |
| SDWt | Chr12:895847 | FASTmrMLM | 7 | 8.1 | 0.00 | 3.96 | 1.05E-09 |  |  |  |
| SDWt | Chr12:2003623 | FASTmrMLM | 3 | 4.4 | 0.10 | 11.88 | 6.44E-06 | Lus10023297 | AT4G18390 | TCP2 |
| SDWt | Chr12:2003623 | FASTmrMLM | 7 | 16.3 | 0.12 | 11.88 | Inf | Lus10023283 | AT2G34640 | PTAC12 |
| SDWt | Chr12:3091575 | FASTmrMLM | 7 | 12.2 | 0.03 | 7.92 | 7.33E-14 |  |  |  |
| SL | Chr12:3690290 | ISIS EM-BLASSO | 3 | 3.3 | 12.58 | 5.94 | Inf | Lus10001638 | AT4G29900 | ACA10 |
|  |  |  |  |  |  |  |  | Lus10001637 | AT5G57090 | PIN2 |
| ARD | Chr12:5504203 | pLARmEB | 3 | 21.5 | 0.13 | 6.93 | Inf | Lus10037827 | AT1G04850 | UBA |
|  |  |  |  |  |  |  |  | Lus10037820 | AT3G17205 | UPL6 |
| ARD | Chr12:5870441 | pLARmEB | 3 | 31.3 | 0.09 | 12.87 | Inf | Lus10037785 | AT5G49520 | WRKY48 |
| RDWt | Chr12:6352775 | pLARmEB | 7 | 59.5 | 0.13 | 7.92 | Inf |  |  |  |
| ARD | Chr12:6618716 | pLARmEB | 3 | 10.1 | 0.02 | 5.94 | 9.04E-12 | Lus10006523 | AT3G16510 | CaLB |
| ARD | Chr12:7572669 | pLARmEB | 3 | 18.7 | 0.04 | 9.9 | Inf |  |  |  |
| SDWt | Chr12:12200657 | mrMLM | 3 | 3.5 | 10.05 | 18.18 | 5.80E-05 | Lus10033041 | AT2G31880 | SOBIR1 |
|  |  |  |  |  |  |  |  | Lus10033043 | AT3G01650 | RGLG1 |
|  |  |  |  |  |  |  |  | Lus10033044 | AT2G26710 | CYP72B1 |
| SDWt | Chr12:17419506 | FASTmrMLM | 7 | 4.6 | 0.00 | 2.97 | 4.52E-06 |  |  |  |
| RDWt | Chr12:20228564 | pKWmEB | 3 | 3.3 | 1.71 | 6.06 | 9.16E-05 | Lus10031478 | AT4G12560 | CPR30 |
| ARD | Chr12:20244330 | ISIS EM-BLASSO | 3 | 3.0 | 0.40 | 7.92 | 1.92E-04 | Lus10031480 | AT4G12560 | CPR30 |
| RDWt | Chr13:1344162 | pLARmEB | 7 | 13.4 | 0.00 | 6.93 | 3.66E-15 |  |  |  |
| RDWt | Chr13:2333295 | pLARmEB | 7 | 13.7 | 0.00 | 4.95 | 1.78E-15 |  |  |  |
| ARD | Chr13:3031249 | pLARmEB | 3 | 16.0 | 0.10 | 7.92 | Inf | Lus10025986 | AT2G18170 | MPK7 |
| RDWt | Chr13:3157081 | pKWmEB | 3 | 4.4 | 0.00 | 12.12 | 6.90E-06 |  |  |  |
| ARD | Chr13:6621308 | pLARmEB | 3 | 10.5 | 0.17 | 6.93 | 3.21E-12 | Lus10034544 | AT1G20160 | ATSBT5.2 |
| SDWt | Chr13:13450469 | FASTmrMLM | 7 | 9.4 | 0.01 | 1.98 | 4.37E-11 |  |  |  |
| SDWt | Chr13:15851979 | LFMM | 7 |  |  |  | 8.06E-06 |  |  |  |
| RDWt | Chr13:15851979 | pLARmEB | 7 | 4.1 | 0.01 | 1.98 | 1.25E-05 |  |  |  |
| SDWt | Chr13:17568553 | FASTmrMLM | 7 | 5.5 | 0.00 | 3.96 | 4.99E-07 |  |  |  |
| ARD | Chr13:17670145 | pLARmEB | 3 | 8.3 | 0.01 | 5.94 | 5.84E-10 | Lus10030723 | AT1G76420 | NAC368 |
| ARD | Chr13:17935031 | FASTmrMLM | 7 | 5.5 | 0.06 | 5.94 | 4.98E-07 |  |  |  |
| SDWt | Chr13:18631902 | FASTmrMLM | 3 | 3.6 | 0.01 | 5.94 | 4.65E-05 | Lus10030921 | AT1G60860 | AGD2 |
|  |  |  |  |  |  |  |  | Lus10030567 | AT4G21200 | GA2OX8 |
| RDWt | Chr14:995061 | pLARmEB | 7 | 24.6 | 0.14 | 8.91 | Inf |  |  |  |
| ARD | Chr14:1596540 | pLARmEB | 3 | 10.0 | 0.10 | 6.93 | 1.08E-11 | Lus10028713 | AT1G34190 | NAC017 |
| ARD | Chr14:2355271 | pLARmEB | 3 | 5.9 | 0.01 | 5.94 | 2.09E-07 | Lus10013393 | AT3G43600 | AO3 |
|  |  |  |  |  |  |  |  | Lus10013387 | AT1G21240 | WAK3 |
|  |  |  |  |  |  |  |  | Lus10013383/4/5 | AT1G21270 | WAK2 |
| ARD | Chr14:3694337 | pLARmEB | 3 | 13.4 | 0.01 | 6.93 | 4.11E-15 | Lus10021401 | AT1G08190 | VAM2,ZIP2 |
| SDWt | Chr14:3695509 | FASTmrMLM | 3 | 4.5 | 0.02 | 9.9 | 5.81E-06 | Lus10021410 | AT2G28190 | CSD2 |
| SDWt | Chr14:3842467 | FASTmrMLM | 7 | 9.7 | 0.00 | 1.98 | 2.11E-11 |  |  |  |
| RDWt | Chr14:4123574 | pKWmEB | 3 | 4.7 | 2.71 | 8.08 | 3.21E-06 | Lus10021466 | AT1G08010 | GATA11 |
| RDWt | Chr14:4123574 | pLARmEB | 7 | 36.3 | 0.36 | 7.92 | Inf | Lus10021467 | AT4G30080 | ARF16 |
| RDWt | Chr14:4767918 | pLARmEB | 7 | 9.9 | 0.15 | 4.95 | 1.52E-11 |  |  |  |
| SDWt | Chr14:8815881 | FASTmrMLM | 7 | 4.2 | 0.00 | 4.95 | 1.19E-05 |  |  |  |
| NWL | Chr14:13363192 | mrMLM | 3 | 4.6 | 9.77 | 9.09 | 4.55E-06 | Lus10032911 | AT3G28860 | MDR1, |
| NWPer | Chr14:13363192 | mrMLM | 3 | 3.7 | 9.05 | 9.09 | 3.99E-05 | Lus10032912 | AT5G24660 | LSU2 |
| NWL | Chr14:13363192 | pLARmEB | 3 | 3.2 | 5.33 | 8.91 | 1.39E-04 |  |  |  |
| NWL | Chr14:13363192 | ISIS EM-BLASSO | 3 | 3.1 | 4.75 | 8.91 | 1.66E-04 |  |  |  |
| NWSA | Chr14:13363192 | mrMLM | 7 | 3.2 | 8.87 | 9.09 | 1.11E-04 |  |  |  |
| NWA | Chr14:13363192 | mrMLM | 7 | 3.9 | 8.44 | 9.09 | 2.52E-05 |  |  |  |
| NWL | Chr14:13363192 | mrMLM | 7 | 3.8 | 10.69 | 9.09 | 2.83E-05 |  |  |  |
| NWPer | Chr14:13363192 | mrMLM | 7 | 3.5 | 9.86 | 9.09 | 5.94E-05 |  |  |  |
| RDWt | Chr14:15018214 | pLARmEB | 7 | 3.4 | 0.01 | 4.95 | 8.25E-05 |  |  |  |
| NWW_Dep | Chr14:15462441 | ISIS EM-BLASSO | 7 | 5.0 | 14.77 | 2.97 | 1.80E-06 |  |  |  |
| RDWt | Chr14:15462441 | pLARmEB | 7 | 6.5 | 0.05 | 2.97 | 4.59E-08 |  |  |  |
| SDWt | Chr14:15952502 | FASTmrMLM | 7 | 4.2 | 0.00 | 4.95 | 1.12E-05 |  |  |  |
| RDWt | Chr14:15956346 | pLARmEB | 7 | 15.6 | 0.06 | 4.95 | Inf |  |  |  |
| SDWt | Chr14:16353008 | FASTmrMLM | 7 | 18.6 | 0.00 | 5.94 | Inf |  |  |  |
| RDWt | Chr14:16353008 | pLARmEB | 7 | 8.1 | 0.12 | 5.94 | 1.02E-09 |  |  |  |
| ARD | Chr14:16745088 | pLARmEB | 3 | 14.4 | 0.02 | 9.9 | 3.33E-16 | Lus10017988 | AT1G48380 | HYP7 |
|  |  |  |  |  |  |  |  | Lus10017984 | AT1G73370 | SUS6 |
| ARD | Chr14:16927209 | pLARmEB | 3 | 8.7 | 0.06 | 5.94 | 2.66E-10 | Lus10005537 | AT5G53950 | CUC2 |
| SDWt | Chr14:16927209 | FASTmrMLM | 7 | 9.6 | 0.00 | 5.94 | 3.17E-11 |  |  |  |
| NWV | Chr14:18171993 | LFMM | 3 |  |  |  | 5.03E-05 | Lus10038981 | AT2G27030 | CAM5 |
| SDWt | Chr14:18171993 | FASTmrMLM | 7 | 3.4 | 0.25 | 10.89 | 7.63E-05 | Lus10038977 | AT1G77760 | NR1 |
| NWW | Chr14:18171993 | LFMM | 7 |  |  |  | 4.00E-05 |  |  |  |
| RDWt | Chr15:41099 | pLARmEB | 7 | 5.6 | 0.03 | 14.85 | 4.17E-07 |  |  |  |
| ARD | Chr15:2735924 | pLARmEB | 3 | 25.5 | 0.20 | 7.92 | Inf | Lus10029410 | AT5G22380 | NAC090 |
| ARD | Chr15:2735924 | FASTmrMLM | 3 | 9.1 | 0.15 | 7.92 | 9.13E-11 |  |  |  |
| RDWt | Chr15:2885976 | pLARmEB | 7 | 73.6 | 0.03 | 5.94 | Inf |  |  |  |
| ARD | Chr15:3479585 | FASTmrMLM | 7 | 7.6 | 0.03 | 3.96 | 3.43E-09 |  |  |  |
| RDWt | Chr15:3849840 | pLARmEB | 7 | 17.4 | 0.01 | 4.95 | Inf |  |  |  |
| SDWt | Chr15:9172034 | FASTmrMLM | 7 | 43.6 | 0.07 | 4.95 | Inf |  |  |  |
| ARD | Chr15:9340878 | pLARmEB | 3 | 6.0 | 0.01 | 5.94 | 1.31E-07 |  |  |  |
| RDWt | Chr15:9525891 | pLARmEB | 7 | 7.5 | 0.07 | 12.87 | 4.66E-09 |  |  |  |
| ARD | Chr15:9795079 | pLARmEB | 3 | 34.5 | 0.14 | 14.85 | Inf | Lus10041216 | AT5G19280 | RAG1 |
| ARD | Chr15:10515587 | pLARmEB | 3 | 22.1 | 0.23 | 9.9 | Inf | Lus10041396 | AT3G61050 | NTMC2T4 |
| MedR | Chr15:10531332 | mrMLM | 3 | 3.8 | 8.52 | 6.06 | 2.65E-05 |  |  |  |
| NWSA | Chr15:11371216 | mrMLM | 3 | 3.6 | 13.23 | 6.06 | 5.03E-05 | Lus10001139 | AT5G63090 | LOB |
| NWA | Chr15:11371216 | mrMLM | 3 | 4.1 | 13.05 | 6.06 | 1.48E-05 | Lus10001137 | AT5G40440 | MKK3 |
| NWW | Chr15:11371216 | mrMLM | 3 | 4.6 | 11.76 | 6.06 | 4.68E-06 |  |  |  |
| NWPer | Chr15:11371216 | mrMLM | 3 | 3.6 | 10.37 | 6.06 | 5.16E-05 |  |  |  |
| NWL | Chr15:11371216 | mrMLM | 3 | 3.0 | 10.21 | 6.06 | 1.79E-04 |  |  |  |
| NWA | Chr15:11371216 | pLARmEB | 3 | 3.8 | 7.15 | 5.94 | 2.58E-05 |  |  |  |
| NWV | Chr15:11371216 | FASTmrMLM | 3 | 3.0 | 6.64 | 5.94 | 1.85E-04 |  |  |  |
| NWV | Chr15:11371216 | ISIS EM-BLASSO | 3 | 3.7 | 6.62 | 5.94 | 3.54E-05 |  |  |  |
| NWV | Chr15:11371216 | pLARmEB | 3 | 3.8 | 6.61 | 5.94 | 2.56E-05 |  |  |  |
| NWA | Chr15:11371216 | ISIS EM-BLASSO | 3 | 3.1 | 6.50 | 5.94 | 1.77E-04 |  |  |  |
| NWSA | Chr15:11371216 | pLARmEB | 3 | 3.6 | 6.50 | 5.94 | 5.03E-05 |  |  |  |
| NWW | Chr15:11371216 | FASTmrMLM | 3 | 3.0 | 5.94 | 5.94 | 1.99E-04 |  |  |  |
| NWL | Chr15:11371216 | pLARmEB | 3 | 3.2 | 5.46 | 5.94 | 1.21E-04 |  |  |  |
| NWL | Chr15:11371216 | ISIS EM-BLASSO | 3 | 3.3 | 5.36 | 5.94 | 1.04E-04 |  |  |  |
| NWA | Chr15:11371216 | FASTmrMLM | 3 | 3.1 | 0.00 | 5.94 | 1.46E-04 |  |  |  |
| NWV | Chr15:11371216 | LFMM | 3 |  |  |  | 6.97E-05 |  |  |  |
| NWSA | Chr15:11371216 | FASTmrMLM | 7 | 3.1 | 7.28 | 5.94 | 1.46E-04 |  |  |  |
| NWW | Chr15:11371216 | FASTmrMLM | 7 | 4.2 | 9.52 | 5.94 | 1.23E-05 |  |  |  |
| NWSA | Chr15:11371216 | ISIS EM-BLASSO | 7 | 3.5 | 7.92 | 5.94 | 5.39E-05 |  |  |  |
| NWA | Chr15:11371216 | ISIS EM-BLASSO | 7 | 3.4 | 8.51 | 5.94 | 7.70E-05 |  |  |  |
| NWL | Chr15:11371216 | ISIS EM-BLASSO | 7 | 3.3 | 6.61 | 5.94 | 1.06E-04 |  |  |  |
| NWPer | Chr15:11371216 | ISIS EM-BLASSO | 7 | 3.6 | 7.47 | 5.94 | 5.23E-05 |  |  |  |
| NWW | Chr15:11371216 | LFMM | 7 |  |  |  | 3.02E-05 |  |  |  |
| NWSA | Chr15:11371216 | mrMLM | 7 | 4.1 | 12.67 | 6.06 | 1.27E-05 |  |  |  |
| NWA | Chr15:11371216 | mrMLM | 7 | 4.4 | 13.67 | 6.06 | 7.20E-06 |  |  |  |
| NWW | Chr15:11371216 | mrMLM | 7 | 4.4 | 14.17 | 6.06 | 7.60E-06 |  |  |  |
| NWA | Chr15:11371216 | pLARmEB | 7 | 4.1 | 9.02 | 5.94 | 1.24E-05 |  |  |  |
| NWL | Chr15:11371216 | pLARmEB | 7 | 3.3 | 6.53 | 5.94 | 1.06E-04 |  |  |  |
| NWPer | Chr15:11371216 | pLARmEB | 7 | 3.3 | 6.87 | 5.94 | 9.27E-05 |  |  |  |
| RDWt | Chr15:13192686 | pLARmEB | 7 | 9.5 | 0.17 | 5.94 | 4.09E-11 |  |  |  |
| ARD | Chr15:14639205 | pLARmEB | 3 | 28.9 | 0.11 | 5.94 | Inf | Lus10014822 | AT3G54220 | SCR,SGR1 |
|  |  |  |  |  |  |  |  | Lus10014821 | AT1G71890 | SUC5 |

^1^Quantitative trait nucleotide chromosome number and position are indicated

^2^ Dataset 3=3K and 7=7K

^3^LOD=logarithm of odds

^4^R^2^=coefficient of determination indicating phenotypic variance explained due to allelic effect

^5^MAF=minor allele frequency

^6^*Arabidobsis thaliana* orthologue for the predicted gene in flax linked to the trait indicated within 100kb up and downstream of the detected QTN.
