# Supplementary material for: The Complex Genetic Architecture of Early Root and Shoot Traits in Flax Revealed by Genome-Wide Association Analyses
Source: Front Plant Sci. 2019 Nov 19;10:1483. doi: 10.3389/fpls.2019.01483 (PMC6878218; doi:10.3389/fpls.2019.01483)
Supplement: Supplementary file 6 [file Table_3.docx]

**Supplementary Table 3.** Associated traits and QTNs detected based on Bonferoni 0.05/n criterion using LFMM from the two datasets

| Trait | QTN^1^ | Dataset^2^ | *P* value | Predicted flax gene | Arabidopsis orthologue | Gene product |
| --- | --- | --- | --- | --- | --- | --- |
| NWW_Dep | Chr1:7339896 | 7 | 5.39E-07 | Lus10029174 | AT3G53710 | AGD6(ARF_GAP domain6) |
| NWL | Chr1:9613871 | 3 | 1.29E-05 | Lus10004206 | AT3G44350 | NAC061 |
| NWPer | Chr1:9613871 | 3 | 7.43E-06 | Lus10004214 | AT3G11620 | ABH |
| NWSA | Chr1:9613871 | 3 | 8.16E-06 | Lus10004225 | AT3G11570 | TBL8 |
| NWV | Chr1:9613871 | 3 | 8.57E-06 |  |  |  |
| NWW | Chr1:9613871 | 3 | 1.52E-05 |  |  |  |
| NWW | Chr1:9613871 | 7 | 2.29E-07 |  |  |  |
| RDWt | Chr1:9613871 | 7 | 8.91E-06 |  |  |  |
| NWW | Chr2:6662071 | 7 | 1.88E-06 |  |  |  |
| NWW_Dep | Chr4:11667975 | 7 | 4.62E-06 |  |  |  |
| RDWt | Chr5:2645287* | 7 | 4.83E-06 |  |  |  |
| NWDep | Chr5:15312783* | 3 | 6.29E-06 |  |  |  |
| NWW_Dep | Chr6:6417516 | 3 | 1.28E-05 | Lus10017778 | AT5G08020 | RPA70B |
| NWW_Dep | Chr6:6427626 | 3 | 1.28E-05 |  |  |  |
| NWW | Chr6:12030162 | 7 | 1.88E-06 |  |  |  |
| NWW | Chr6:16928785 | 7 | 2.68E-06 |  |  |  |
| NWL | Chr9:19061342 | 3 | 4.27E-06 | Lus10024833 | AT2G38290 | AMT2,AMT2;1 |
| NWPer | Chr9:19061342 | 3 | 2.43E-06 | Lus10024853 | AT1G04240 | IAA3,SHY2 |
| NWW | Chr9:19061342 | 3 | 1.28E-05 |  |  |  |
| NWPer | Chr9:19061342* | 7 | 5.24E-06 |  |  |  |
| NWW | Chr9:19061342 | 7 | 3.76E-07 |  |  |  |
| NWW_Dep | Chr9:19061342 | 7 | 1.62E-06 |  |  |  |
| NWW_Dep | Chr11:5123720 | 7 | 1.13E-07 |  |  |  |
| SDWt | Chr11:8154007* | 3 | 7.74E-06 | Lus10036374 | AT4G00350 | MATE efflux |
| SDWt | Chr11:8176149 | 3 | 2.24E-06 | Lus10036372 | AT1G63800 | UBC5 |
| SDWt | Chr11:8176149* | 7 | 1.5E-06 |  |  |  |

^1^Quantitative trait nucleotide; chromosome number and position are indicated; *indicates QTN detected by at least one multi-locus method being associated with the same corresponding trait.

^2^ Dataset 3=3K and 7=7K
